# Supplementary material for: Bacterial sexually transmitted infections and related antibiotic use among individuals eligible for doxycycline post-exposure prophylaxis in the United States
Source: Nat Commun. 2025 Oct 16;16:9206. doi: 10.1038/s41467-025-64261-w (PMC12532492; doi:10.1038/s41467-025-64261-w)
Supplement: Supplementary file 1 — Supplementary Information [file 41467_2025_64261_MOESM1_ESM.pdf]

Supporting Information

Bacterial sexually transmitted infections and related antibiotic use among individuals eligible for doxycycline post-exposure prophylaxis in the United States

Anna M. Parker[1], Jennifer J. Chang[2], Ligong Chen[3], Laura M. King[1], Sandra I. McCoy[1,4], Joseph A. Lewnard[1,5,6]†,*, Katia J. Bruxvoort[7]†

1. Division of Epidemiology, School of Public Health, University of California, Berkeley, Berkeley, California 94720, United States
2. Department of Infectious Diseases, Los Angeles Medical Center, Southern California Permanente Medical Group, Los Angeles, California, United States
3. Perisphere Real World Evidence, Austin, Texas 78751, United States
4. Gilead Sciences, Inc., Foster City, California 94404, United States
5. Division of Infectious Diseases & Vaccinology, School of Public Health, University of California, Berkeley, Berkeley, California 94720, United States
6. Center for Computational Biology, College of Computing, Data Science, and Society, University of California, Berkeley, Berkeley, California 94720, United States
7. Department of Epidemiology, University of Alabama at Birmingham, 1665 University Boulevard, Birmingham, AL, 35233, United States

† These authors jointly supervised the work.

* Address for correspondence:
Joseph Lewnard
2121 Berkeley Way, Room 5410
Berkeley, California 94122
United States
Email: jLewnard@berkeley.edu
Tel.: +1 (510) 664-4050

Contents of this supplement

Item	Title	Page
Text S1	Supplemental methods.	3
Table S1	Demographic characteristics of the study population, by risk stratum.	5
Table S2	Incidence rate of sexually transmitted infection diagnoses among males and transgender individuals stratified by year and over the entire study period.	6
Table S3	Incidence rate of gonorrhea diagnoses among males and transgender individuals stratified by year.	7
Table S4	Incidence rate of chlamydia diagnoses among males and transgender individuals stratified by year.	8
Table S5	Incidence rate of syphilis diagnoses among males and transgender individuals stratified by year.	9
Table S6	Incidence rate of oral and injection antibiotic fills related to sexually transmitted infection diagnoses among males and transgender individuals aged 18 to 24 years from 2017 to 2019 in the United States.	10
Table S7	Incidence rate of oral and injection antibiotic fills related to sexually transmitted infection diagnoses among males and transgender individuals aged 25 to 34 years from 2017 to 2019 in the United States.	11
Table S8	Incidence rate of oral and injection antibiotic fills related to sexually transmitted infection diagnoses among males and transgender individuals aged 35 to 44 years from 2017 to 2019 in the United States.	12
Table S9	Incidence rate of oral and injection antibiotic fills related to sexually transmitted infection diagnoses among males and transgender individuals aged 45 to 65 years from 2017 to 2019 in the United States.	13
Table S10	Incidence rate of oral and injection antibiotic fills related to gonorrhea diagnoses among males and transgender individuals from 2017 to 2019 in the United States.	14
Table S11	Incidence rate of oral and injection antibiotic fills related to chlamydia diagnoses with and without a gonorrhea co-infection among males and transgender individuals from 2017 to 2019 in the United States.	15

Table S12	Incidence rate of oral and injection antibiotic fills related to syphilis diagnoses among males and transgender individuals from 2017 to 2019 in the United States.	16
Table S13	Incidence rate difference of sexually transmitted infection diagnoses among males and transgender individuals with 100% doxycycline postexposure prophylaxis (doxyPEP) uptake.	17
Table S14	Estimates of incidence rate difference in standardized tetracycline fills with 100% doxycycline postexposure prophylaxis (doxyPEP) uptake utilizing alternative estimates of sexual encounter frequencies.	18
Table S15	Incidence rate difference of oral and injection antibiotic fills related to sexually transmitted infection diagnoses among males and transgender individuals with doxycycline postexposure prophylaxis having no effect on gonorrhea.	19
Table S16	Estimated increase in standardized tetracycline fills per sexually transmitted infection diagnosis prevented.	20
Table S17	Codes used to identify transgender persons.	21
Table S18	National Drug Codes used to identify Emtricitabine/Tenofovir use.	22
Table S19	Codes used to identify HIV infection.	23
Table S20	Codes used to identify HIV-associated opportunistic infections.	24
Table S21	Codes used to identify Hepatitis B infection.	25
Table S22	Codes used to identify needlestick exposure.	26
Table S23	Codes used to identify gonococcal infection.	27
Table S24	Codes used to identify chlamydial infection.	28
Table S25	Codes used to identify syphilis infection.	29
Table S26	Healthcare Common Procedure Coding System (HCPCS) codes used to identify injectable antibiotics related to sexually transmitted infections.	30
Table S27	Oral and injection antibiotics with first- or second-line classification used to treat sexually transmitted infections in the United States 2017 to 2019.	31
Table S28	Estimated annual rates of new or casual unprotected anal sex partnerships among MSM according to risk characteristics.	32
Supplemental references		33

Text S1: Supplemental methods.

To estimate the frequency with which MSM in differing risk strata may use doxyPEP, we used data from previous studies quantifying rates of unprotected anal sex partnerships with new or casual partners among MSM and studies measuring associations between MSM's recent numbers of such partnerships and their risk of bacterial STI diagnoses.

Reference population

We considered the sample enrolled in the ARTnet study as a reference population for quantifying rates of sexual partnership formation.[1,2] The sample for this study was recruited online among MSM between July 2017 and January 2019 directly after participation in the American Men's Internet Study,[3] an annual online behavioral survey recruiting MSM via email blasts and banner advertisements on websites or mobile phone applications targeting differing audiences (gay social networking, gay general interest, general social networking, and geospatial social networking). Consistency of sexually-transmitted infection incidence within this study sample and US surveillance data suggest risk characteristics of the population recruited in the ARTnet are representative of those of MSM in general. Specifically, self-reported HIV prevalence within the study sample was 10.8%, comparable to the estimate of 11.1% prevalence of diagnosed HIV infection among all US MSM.[4] Gonorrhea incidence within the sample was 5.07 cases/100 person-years, comparable to the estimate of 5.17 cases/100 person-years among MSM.[5] Additionally, prior comparative studies have reported that sexual risk behaviors are similar in MSM samples recruited through such online surveys and those recruited via physical venue-based sampling [6].

To account for the right-tailed shape of the distribution of individuals' annual reported partnerships, we considered this count to follow a negative binomial distribution, $Z_j \sim \text{Negative Binomial}(p_j, r_j)$ within strata (j) comprised of MSM using PrEP, MSM with HIV, and the general MSM population. We fit parameters p_j and r_j via least-squares fitting against reported distribution quantiles for each stratum within ARTnet study publications.[1,2] We sampled from the fitted distributions of the number of partnerships among pseudo-populations of 1 million individuals for each stratum to generate a random variable of individuals' annual partnership counts, $Z(i)$.

Partnership counts by STI history

We identified three US-based studies reporting data on STI risk and numbers of partnerships over differing recall periods among MSM.[7–9] The cohort study we used to parameterize our primary analysis model[8] reported annual incidence rates of gonorrhea and chlamydia urethral or rectal infections across strata of individuals with 1, 2-5, 6-10, 11-50, or ≥50 partners over a 6-month observation period. Using $Z(i)/2$ to measure individuals' expected number of partnerships over a 6-month period within the pseudo-population, we generated individual-specific rate parameters for STI diagnoses $\lambda(i)$ corresponding to their 6-month history of sexual partnerships:

$$\lambda(i) = \begin{cases} 0.0658 & \text{if } Z(i)/2 < 2 \\ 0.1192 & \text{if } 2 \le Z(i)/2 < 6 \\ 0.1918 & \text{if } 6 \le Z(i)/2 < 10 \\ 0.244 & \text{if } 10 \le Z(i)/2 < 50 \\ 0.3385 & \text{if } Z(i)/2 \ge 50 \end{cases}$$

In turn, we sampled the number of STI diagnoses among individuals over a given year for our pseudo-population as

$$Y(i) \sim \text{Pois}(\lambda(i)).$$

We used the resulting draws of Y to obtain conditional distributions of individuals' annual number of partnerships, Z, for strata with ≥1, ≥2, and ≥3 STI diagnoses in the prior year ($Y \ge 1$, $Y \ge 2$, and $Y \ge 3$; **Table S28**).

A second study reported numbers of partnerships in the preceding 12 months among MSM testing positive for gonorrhea or chlamydia to those testing negative in a US national sample.[7] To recover distributions of encounter rates among MSM expected to experience ≥1, ≥2, or ≥3 bacterial STIs annually, we first parameterized the relative risk of STI diagnosis (assumed to be equivalent to the incidence rate ratio of STI diagnoses) per additional sexual encounter. We fit Gamma distributions to reported 50%, 25%, and 75% quantiles of individuals' partnerships per year among those who tested positive for gonorrhea or chlamydia and those testing negative for both to recover rates of sexual partnership formation:

$$Z^*_{\text{STI}=1} \sim \text{Gamma}(\alpha_1, \beta_1)$$

$$Z^*_{\mathrm{STI}=0} \sim \mathrm{Gamma}(\alpha_0, \beta_0)$$

We sampled one million draws from the distribution of each rate, and used these draws from the resulting pseudo-population of individuals with or without STI diagnoses to estimate the relative risk via Poisson regression. We used the resulting regression coefficients to project individual-specific rates of STI diagnosis, $\lambda(i)$, as a function of their rate of partnership formation for the full pseudo-population,

$$\lambda(i) = \exp[\beta_0 + \beta_1 Z(i)].$$

In turn, we defined individuals' number of STI diagnoses in a given year, $Y(i)$, as a Poisson random variable parameterized by the individual-specific rate $\lambda(i)$,

$$Y(i) \sim \mathrm{Pois}\big(\lambda(i)\big).$$

Finally, a third cohort study[9] presented a continuous relationship of cumulative risk of a gonorrhea or chlamydia diagnosis over a 6-month period with respect to their number of casual partners over the same period. We digitized figures presenting this relationship to define the joint distribution of $\lambda/2$ with respect to $Z/2$, and sampled individuals' number of STI diagnoses in a given 12-month period as

$$Y(i) \sim \mathrm{Pois}\big(\lambda(i)\big).$$

Table S1: Demographic characteristics of the study population, by risk stratum.

Characteristics		Proportion (%) of study population														
		PLWH			*PrEP recipient*			*STI history*								
								≥1 STI			≥2 STIs			≥3 STIs		
Year		*2017*	*2018*	*2019*	*2017*	*2018*	*2019*	*2017*	*2018*	*2019*	*2017*	*2018*	*2019*	*2017*	*2018*	*2019*
Total, *N*		8095	10122	11011	10679	12480	15384	5621	6688	7609	825	1041	1249	104	175	212
Sex[1]																
	Female	0.1	0.1	0.2	0.1	0.2	0.3	0.1	0.1	0.3	0.1	0.1	0.2	0	0	0
	Male	99.9	99.9	99.8	99.9	99.8	99.7	99.9	99.9	99.7	99.9	99.9	99.8	100	100	100
Transgender[2]																
	Yes	0.2	0.3	0.5	0.3	0.6	0.7	0.3	0.3	0.4	0.2	0.4	0.5	0	0.6	0.5
	No	99.8	99.7	99.5	99.7	99.4	99.3	99.7	99.7	99.6	99.8	99.6	99.5	100	99.4	99.5
STIs in calendar year																
	0	93.5	93.3	93.4	92.9	91.5	91.4	0	0	0	0	0	0	0	0	0
	1	5.2	5.5	5.4	5.6	6.8	6.6	85.3	84.4	83.6	0	0	0	0	0	0
	2	0.9	0.9	0.9	1.2	1.4	1.5	12.8	12.9	13.6	87.4	83.2	83	0	0	0
	3	0.2	0.2	0.2	0.2	0.3	0.3	1.3	1.9	1.9	8.6	12.1	11.4	68.3	72	67
	>4	0.1	0.1	0.1	0.1	0.1	0.1	0.6	0.7	0.9	4	4.7	5.6	31.7	28	33
Age group																
	18-24 years	4.5	4.1	3.8	7.5	9.1	9.7	35.1	33.9	31.6	30.3	31.2	28.3	22.1	22.3	24.1
	25-34 years	12.5	13.8	13.9	22	26.4	28.8	25.4	26.6	28.3	29	28	29.1	26.9	32.6	28.3
	35-44 years	18.2	18.7	18.1	23.7	24.4	25	18	18.1	19.3	18.8	21.7	21.9	20.2	21.7	22.6
	45-65 years	64.7	63.4	64.2	46.8	40.1	36.6	21.5	21.5	20.8	21.9	19	20.8	30.8	23.4	25
Region																
	West South Central	11.3	8.4	9.5	10.6	9.3	10.2	12.5	9.3	9.6	10.5	7.2	8.5	12.5	8.6	5.7
	Mountain	4.1	4.1	4.4	5.4	5.3	5.6	4.2	4.5	4.2	4.5	3.8	4	4.8	3.4	4.7
	East South Central	6.8	4.4	4.1	3.5	2.1	2.6	5.1	4.3	4.2	3.6	4	3.4	2.9	2.3	2.4
	Middle Atlantic	16	12.3	15.8	17.4	14.3	20.8	17.4	13.3	20.6	20	13.9	22.7	22.1	13.1	25.5
	South Atlantic	33.8	29.9	30.3	23.3	20.4	22	29.1	24.1	24.3	27.6	28	27.7	23.1	28.6	26.4
	West North Central	3.5	3.1	2.7	3	3.1	2.7	3.5	3.7	3.4	3.4	3.8	1.9	1.9	3.4	0.9
	East North Central	9.2	10.1	10.1	9.9	12.1	11.8	11.4	12.8	11.1	9	10.8	8.8	3.8	9.1	7.1
	Pacific	11.7	11.3	8	21.7	21	15.1	12.9	13.3	11.2	16.8	14.5	13.3	26	18.9	16
	New England	2.9	2.5	2.4	4.4	4.2	3.9	3.4	3.1	3.4	4.1	3	3	2.9	2.3	2.4
	Missing or Unknown	0.6	13.9	12.8	0.9	8.2	5.4	0.4	11.7	8	0.4	10.9	6.8	0	10.3	9

STIs: Sexually-transmitted infections (gonorrhea, chlamydia, syphilis).
1: Indicates the last sex registered in medical claims data.
2: Transgender individuals are those with a diagnosis or medical procedure indicative of transgender status.

Table S2: Incidence rate of sexually transmitted infection diagnoses among males and transgender individuals stratified by year.

Cohort		Incidence rate of STIs per 100-person years (95% CI)		
		2017	*2018*	*2019*
PLWH[1]				
	All	7.6 (6.9, 8.4)	8.1 (7.4, 8.8)	8.6 (7.9, 9.3)
	≥1 STI in year prior	30.0 (25.1, 35.9)	31.4 (26.4, 37.2)	37.5 (32.2, 44.0)
	≥2 STIs in year prior	50.5 (36.6, 69.7)	55.5 (41.9, 73.1)	74.2 (56.7, 98.0)
	≥3 STIs in year prior	76.9 (47.4, 126.6)	60.5 (40.1, 92.2)	136.3 (91.2, 203.0)
PrEP use[2]				
	All	10.2 (9.5, 10.9)	10.9 (10.3, 11.7)	11.1 (10.4, 11.7)
	≥1 STI in year prior	34.6 (30.0, 39.7)	33.5 (29.7, 38)	37.7 (33.7, 42.3)
	≥2 STIs in year prior	49.3 (37.7, 64.4)	59.0 (48.1, 72.6)	64.1 (52.8, 78.0)
	≥3 STIs in year prior	51.7 (30.1, 87.0)	78.0 (53.7, 113.8)	97.5 (69.0, 138.2)
Active PrEP use[3]				
	All	11.7 (10.8, 12.7)	12.2 (11.4, 13.0)	12.4 (11.7, 13.2)
	≥1 STI in year prior	39.3 (34.3, 45.2)	39.7 (35.5, 44.4)	38.8 (34.7, 43.3)
	≥2 STIs in year prior	57.9 (44.9, 74.5)	66.2 (55.2, 79.3)	66.8 (55.7, 80.1)
	≥3 STIs in year prior	60.7 (38.7, 94.6)	81.1 (57.4, 114.5)	102.4 (74.6, 140.5)
Consistent PrEP use[4]				
	All	12.0 (11.1, 13.1)	13.1 (12.1, 14.1)	13.5 (12.6, 14.5)
	≥1 STI in year prior	42.0 (36.2, 48.8)	43.5 (38.4, 49.3)	43.7 (38.7, 49.4)
	≥2 STIs in year prior	57.7 (42.8, 77.6)	68.6 (55.7, 84.4)	76.9 (63.5, 93.0)
	≥3 STIs in year prior	69.9 (39, 124.9)	116.7 (84.2, 161.5)	116.6 (85.3, 159.2)
STI history				
	≥1 STI in year prior	16.7 (15.5, 18.1)	17.6 (16.3, 18.9)	18.7 (17.4, 20.1)
	≥2 STIs in year prior	35.5 (30.6, 41.3)	37.7 (33.0, 43)	43.8 (38.7, 49.6)
	≥3 STIs in year prior	83.6 (63.6, 110.0)	73.0 (57.7, 92.4)	89.6 (72.2, 111.3)

CI: Confidence interval; STIs: Sexually-transmitted infections (gonorrhea, chlamydia, syphilis).
[1]People living with HIV (PLWH).
[2]PrEP use: filled ≥1 PrEP prescription in the previous year.
[3]Active PrEP use: ≥1 PrEP prescription in the previous three months.
[4]Consistent PrEP use: ≥1 PrEP prescription in the previous three months and ≥3 PrEP fills in the previous year.

Table S3: Incidence rate of gonorrhea diagnoses among males and transgender individuals stratified by year.

Cohort		Incidence rate of gonorrhea diagnoses per 100-person years (95% CI)		
		2017	*2018*	*2019*
PLWH[1]				
	All	3.0 (2.6, 3.5)	3.4 (3.0, 3.8)	3.5 (3.1, 3.9)
	≥1 STI in year prior	15.1 (11.7, 19.5)	15.7 (12.8, 19.4)	18.1 (15.0, 21.9)
	≥2 STIs in year prior	28.3 (19.0, 42.0)	28.1 (19.6, 40.5)	74.8 (48.7, 114.9)
	≥3 STIs in year prior	38.5 (20.9, 72.5)	33.3 (19.2, 57.3)	136.3 (91.2, 203.0)
PrEP use[2]				
	All	5.5 (5.0, 6.0)	6.3 (5.8, 6.8)	5.9 (5.5, 6.4)
	≥1 STI in year prior	20.2 (17.1, 23.9)	21.6 (18.6, 25.1)	22.0 (19.2, 25.1)
	≥2 STIs in year prior	31.8 (24.1, 41.9)	40.9 (32.2, 52)	37.3 (29.8, 46.3)
	≥3 STIs in year prior	29.7 (16.6, 52.9)	49.9 (31, 81)	56.8 (39.9, 80.6)
Active PrEP use[3]				
	All	6.6 (6.0, 7.2)	7.1 (6.5, 7.7)	6.7 (6.2, 7.2)
	≥1 STI in year prior	23.6 (20.1, 27.8)	25.5 (22.3, 29.3)	22.4 (19.7, 25.6)
	≥2 STIs in year prior	34.2 (26.0, 45.1)	43.4 (35.2, 53.4)	39.2 (31.7, 48.4)
	≥3 STIs in year prior	39.4 (24.4, 63.2)	52.7 (35.3, 78.4)	64.4 (46.3, 89.6)
Consistent PrEP use[4]				
	All	6.7 (6.0, 7.5)	7.7 (7, 8.4)	7.3 (6.7, 7.9)
	≥1 STI in year prior	25.7 (21.5, 30.7)	27.5 (23.6, 32)	24.2 (20.8, 28.1)
	≥2 STIs in year prior	33.6 (24, 46.8)	43.6 (34.1, 55.8)	45.2 (36.2, 56.4)
	≥3 STIs in year prior	37 (20.4, 67.1)	72.6 (48.8, 107.8)	70.3 (49.7, 99.3)
STI history				
	≥1 STI in year prior	7.6 (6.8, 8.5)	8.8 (8, 9.7)	9.0 (8.2, 9.8)
	≥2 STIs in year prior	16.7 (13.8, 20.3)	19.3 (16.3, 22.8)	22.4 (19.3, 26)
	≥3 STIs in year prior	36.5 (25.6, 52)	33.7 (24.8, 46)	46.6 (37, 59.4)

CI: Confidence interval; STIs: Sexually-transmitted infections (gonorrhea, chlamydia, syphilis).
[1]People living with HIV (PLWH).
[2]PrEP use: filled ≥1 PrEP prescription in the previous year.
[3]Active PrEP use: ≥1 PrEP prescription in the previous three months.
[4]Consistent PrEP use: ≥1 PrEP prescription in the previous three months and ≥3 PrEP fills in the previous year.

Table S4: Incidence rate of chlamydia diagnoses among males and transgender individuals stratified by year.

Cohort		Incidence rate of chlamydia diagnoses per 100-person years (95% CI)		
		2017	*2018*	*2019*
PLWH[1]				
	All	1.6 (1.3, 1.9)	1.9 (1.6, 2.2)	2 (1.7, 2.3)
	≥1 STI in year prior	7.1 (5.1, 9.9)	8.4 (6.1, 11.4)	11.3 (8.6, 15.1)
	≥2 STIs in year prior	15.3 (9, 25.6)	17.2 (11.5, 25.6)	44.2 (22.6, 86.9)
	≥3 STIs in year prior	27 (11.7, 62.9)	15.2 (6.7, 33.9)	136.3 (91.2, 203)
PrEP use[2]				
	All	2.8 (2.5, 3.2)	3 (2.6, 3.4)	3.3 (3, 3.7)
	≥1 STI in year prior	10.5 (8.2, 13.4)	7.5 (5.9, 9.7)	11.8 (9.8, 14.3)
	≥2 STIs in year prior	16.2 (10.2, 25.8)	14.5 (9.6, 21.9)	21.4 (16, 28.9)
	≥3 STIs in year prior	22.3 (7.6, 65)	24 (14.1, 41.6)	35.1 (21.1, 59.5)
Active PrEP use[3]				
	All	3.1 (2.7, 3.6)	3.3 (2.9, 3.7)	3.7 (3.4, 4.1)
	≥1 STI in year prior	12.0 (9.5, 15.2)	9.5 (7.7, 11.7)	12.1 (10.2, 14.5)
	≥2 STIs in year prior	20.2 (13.5, 30.2)	17.8 (12.8, 24.9)	21.5 (16.3, 28.3)
	≥3 STIs in year prior	18.1 (7.3, 44.2)	24.2 (14.0, 42.0)	30.9 (19.0, 50.2)
Consistent PrEP use[4]				
	All	3.2 (2.8, 3.7)	3.5 (3.1, 4)	4.1 (3.6, 4.6)
	≥1 STI in year prior	13.1 (10.1, 17)	11.9 (9.4, 15)	15.4 (12.8, 18.5)
	≥2 STIs in year prior	20.6 (12.7, 33.5)	22.1 (15.1, 32.2)	26.2 (19.6, 35)
	≥3 STIs in year prior	24.6 (7.7, 77.9)	44 (25.8, 75.1)	43.1 (27.7, 67.1)
STI history				
	≥1 STI in year prior	7.3 (6.5, 8.1)	6.7 (6.0, 7.5)	7.5 (6.8, 8.3)
	≥2 STIs in year prior	16.6 (13.5, 20.4)	15.5 (12.9, 18.7)	17.2 (14.5, 20.4)
	≥3 STIs in year prior	41.2 (26.7, 64.1)	34.3 (24.8, 47.8)	36.3 (26.6, 49.3)

CI: Confidence interval; STIs: Sexually-transmitted infections (gonorrhea, chlamydia, syphilis).
[1]People living with HIV (PLWH).
[2]PrEP use: filled ≥1 PrEP prescription in the previous year.
[3]Active PrEP use: ≥1 PrEP prescription in the previous three months.
[4]Consistent PrEP use: ≥1 PrEP prescription in the previous three months and ≥3 PrEP fills in the previous year.

Table S5. Incidence rate of syphilis diagnoses among males and transgender individuals stratified by year

Cohort		Incidence rate of syphilis diagnoses per 100-person years (95% CI)		
		2017	*2018*	*2019*
PLWH[1]				
	All	3.0 (2.6, 3.4)	2.9 (2.6, 3.3)	3.1 (2.8, 3.5)
	≥1 STI in year prior	7.8 (5.8, 10.5)	7.2 (5.5, 9.4)	8.1 (6.3, 10.4)
	≥2 STIs in year prior	7.0 (3.5, 14.3)	10.1 (6.1, 17.1)	16.6 (7.9, 34.3)
	≥3 STIs in year prior	11.7 (4.0, 33.9)	12.1 (4.8, 31.1)	136.3 (91.2, 203.0)
PrEP use[2]				
	All	2.0 (1.7, 2.2)	1.7 (1.5, 1.9)	1.8 (1.6, 2.1)
	≥1 STI in year prior	3.9 (2.7, 5.5)	4.4 (3.3, 5.9)	3.9 (3.0, 5.2)
	≥2 STIs in year prior	1.3 (0.3, 5.2)	3.6 (1.9, 7.2)	5.5 (3.5, 8.7)
	≥3 STIs in year prior	0 (0, 0)	4.0 (1.0, 15.6)	5.4 (2.1, 14.1)
Active PrEP use[3]				
	All	2.0 (1.7, 2.4)	1.8 (1.5, 2.0)	2.0 (1.8, 2.3)
	≥1 STI in year prior	3.7 (2.5, 5.5)	4.6 (3.5, 6.1)	4.2 (3.3, 5.3)
	≥2 STIs in year prior	3.4 (1.5, 7.9)	4.9 (2.9, 8.3)	6.1 (4.1, 9.0)
	≥3 STIs in year prior	3.3 (0.8, 13.2)	4.1 (1.2, 13.9)	7.0 (3.6, 13.6)
Consistent PrEP use[4]				
	All	2.1 (1.8, 2.5)	1.9 (1.6, 2.2)	2.2 (1.9, 2.5)
	≥1 STI in year prior	3.2 (2.0, 5.2)	4.2 (3.0, 5.9)	4.1 (3.0, 5.6)
	≥2 STIs in year prior	3.4 (1.3, 9.0)	2.8 (1.2, 6.7)	5.5 (3.2, 9.3)
	≥3 STIs in year prior	8.2 (2.1, 32.4)	0 (0, 0)	3.2 (0.8, 12.7)
STI history				
	≥1 STI in year prior	1.9 (1.6, 2.3)	2.1 (1.8, 2.4)	2.3 (1.9, 2.6)
	≥2 STIs in year prior	2.2 (1.4, 3.5)	2.9 (2.1, 4.1)	4.2 (3.2, 5.4)
	≥3 STIs in year prior	5.8 (2.7, 12.5)	5.1 (2.7, 9.7)	6.6 (4.0, 10.9)

CI: Confidence interval; STIs: Sexually-transmitted infections (gonorrhea, chlamydia, syphilis).
[1]People living with HIV (PLWH).
[2]PrEP use: filled ≥1 PrEP prescription in the previous year.
[3]Active PrEP use: ≥1 PrEP prescription in the previous three months.
[4]Consistent PrEP use: ≥1 PrEP prescription in the previous three months and ≥3 PrEP fills in the previous year.

Table S6: Incidence rate of oral and injection antibiotic fills related to sexually transmitted infection diagnoses among males and transgender individuals aged 18 to 24 years from 2017 to 2019 in the United States.

**Cohort**		**Incidence of antibiotic fills related to STIs per 100-person years (95% CI)**					
		Cephalosporins	*Macrolides*	*Penicillins*	*Tetracyclines*	*Quinolones*	*Aminoglycosides*
PLWH[1]							
	All	4.92 (3.4, 7.12)	3.32 (1.93, 5.8)	5.91 (4.02, 8.68)	2.82 (1.39, 5.84)	0.12 (0.02, 0.87)	0 (0, 0)
	≥ 1 STI year prior	5.9 (2.37, 14.65)	5.05 (1.45, 17.73)	4.21 (1.15, 15.29)	5.05 (1.37, 20.26)	0 (0, 0)	0 (0, 0)
	≥ 2 STI year prior	6.82 (0.99, 47.02)	10.23 (1.48, 70.53)	3.41 (0.49, 23.51)	0 (0, 0)	0 (0, 0)	0 (0, 0)
	≥ 3 STI year prior	0 (0, 0)	0 (0, 0)	0 (0, 0)	0 (0, 0)	0 (0, 0)	0 (0, 0)
PrEP[2]							
	All	5.38 (4.49, 6.45)	4.17 (3.14, 5.56)	2.71 (1.95, 3.75)	1.62 (0.93, 2.82)	0.12 (0.04, 0.39)	0 (0, 0)
	≥ 1 STI year prior	12.32 (8.62, 17.59)	10.67 (6.14, 18.59)	9.85 (5.98, 16.22)	4.5 (1.51, 13.51)	0 (0, 0)	0 (0, 0)
	≥ 2 STI year prior	16.61 (9.23, 29.85)	22.12 (10.31, 50.6)	7.37 (2.86, 18.97)	1.83 (0.26, 12.81)	0 (0, 0)	0 (0, 0)
	≥ 3 STI year prior	28.44 (12.39, 65.16)	14.13 (2.13, 93.64)	7.07 (1.06, 46.82)	0 (0, 0)	0 (0, 0)	0 (0, 0)
Active PrEP[3]							
	All	6.23 (5.12, 7.57)	5.18 (3.88, 6.97)	2.37 (1.68, 3.33)	1.87 (1.03, 3.4)	0.16 (0.05, 0.51)	0.05 (0.01, 0.39)
	≥ 1 STI year prior	15 (10.6, 21.21)	12.57 (7.51, 21.25)	7.73 (4.29, 13.91)	5.3 (1.83, 15.99)	0 (0, 0)	0 (0, 0)
	≥ 2 STI year prior	16.87 (8.83, 32.18)	12.62 (4.4, 37.54)	8.42 (3.21, 22.02)	4.19 (0.6, 29.34)	0 (0, 0)	0 (0, 0)
	≥ 3 STI year prior	8.25 (1.41, 47.97)	16.49 (2.48, 109.41)	8.24 (1.14, 59.39)	0 (0, 0)	0 (0, 0)	0 (0, 0)
Consistent PrEP[4]							
	All	7.56 (5.95, 9.6)	5.83 (4, 8.6)	2.59 (1.67, 4.02)	1.94 (0.84, 4.47)	0.21 (0.05, 0.86)	0 (0, 0)
	≥ 1 STI year prior	16.18 (10.54, 24.82)	10.77 (5.42, 21.97)	3.07 (1.19, 7.91)	4.59 (1.02, 21.45)	0 (0, 0)	0 (0, 0)
	≥ 2 STI year prior	20.21 (10.26, 39.77)	11.51 (3.42, 44.56)	2.87 (0.42, 19.72)	2.87 (0.4, 20.29)	0 (0, 0)	0 (0, 0)
	≥ 3 STI year prior	10.71 (1.96, 58.44)	21.39 (3.29, 139.19)	0 (0, 0)	0 (0, 0)	0 (0, 0)	0 (0, 0)
STI							
	≥ 1 STI year prior	2.57 (2.08, 3.18)	3.25 (2.57, 4.11)	1.29 (0.92, 1.8)	1.25 (0.84, 1.9)	0.04 (0.01, 0.15)	0.18 (0.04, 0.9)
	≥ 2 STI year prior	4.6 (3.17, 6.68)	8.12 (5.32, 12.38)	1.22 (0.59, 2.5)	1.62 (0.77, 3.58)	0 (0, 0)	0 (0, 0)
	≥ 3 STI year prior	11.35 (5.78, 22.26)	7.55 (2.57, 23)	2.51 (0.65, 9.71)	0 (0, 0)	0 (0, 0)	0 (0, 0)

CI: Confidence interval; STIs: Sexually-transmitted infections (gonorrhea, chlamydia, syphilis).
[1]People living with HIV (PLWH).
[2]PrEP use: filled ≥1 PrEP prescription in the previous year.
[3]Active PrEP use: ≥1 PrEP prescription in the previous three months.
[4]Consistent PrEP use: ≥1 PrEP prescription in the previous three months and ≥3 PrEP fills in the previous year.

/

Table S7: Incidence rate of oral and injection antibiotic fills related to sexually transmitted infection diagnoses among males and transgender individuals aged 25 to 34 years from 2017 to 2019 in the United States.

**Cohort**		**Incidence of antibiotic fills related to STIs per 100-person years (95% CI)**					
		Cephalosporins	*Macrolides*	*Penicillins*	*Tetracyclines*	*Quinolones*	*Aminoglycosides*
PLWH[1]							
	All	4.71 (3.94, 5.63)	3.43 (2.68, 4.41)	10.96 (9.48, 12.67)	2.2 (1.47, 3.32)	0.1 (0.04, 0.27)	0 (0, 0)
	≥ 1 STI year prior	12.11 (9.16, 16.01)	7.93 (4.85, 13.4)	22.77 (17.37, 29.83)	5.21 (2.59, 10.71)	0.21 (0.03, 1.47)	0 (0, 0)
	≥ 2 STI year prior	16.76 (10.44, 26.88)	7.53 (3.58, 18.82)	20.95 (12.96, 33.83)	8.35 (2.69, 26.91)	0 (0, 0)	0 (0, 0)
	≥ 3 STI year prior	35.88 (19.52, 65.85)	15.92 (6.47, 39.12)	23.89 (10.32, 55.2)	0 (0, 0)	0 (0, 0)	0 (0, 0)
PrEP[2]							
	All	6.53 (5.97, 7.15)	4.96 (4.37, 5.63)	3.56 (3.07, 4.13)	1.79 (1.37, 2.35)	0.06 (0.03, 0.13)	0.06 (0.02, 0.15)
	≥ 1 STI year prior	17.06 (14.26, 20.4)	11.22 (8.65, 14.62)	8.02 (5.83, 11.02)	4.19 (2.41, 7.37)	0.18 (0.05, 0.72)	0.09 (0.01, 0.64)
	≥ 2 STI year prior	22.09 (15.95, 30.58)	11.82 (7.18, 19.85)	9.06 (5.06, 16.2)	4.71 (1.42, 16.74)	0 (0, 0)	0 (0, 0)
	≥ 3 STI year prior	34.99 (21.38, 57.22)	17.46 (7.87, 44.55)	12.22 (5.04, 29.55)	6.96 (1.48, 34.18)	0 (0, 0)	0 (0, 0)
Active PrEP[3]							
	All	7.56 (6.89, 8.3)	5.96 (5.22, 6.81)	3.7 (3.16, 4.32)	1.89 (1.41, 2.54)	0.05 (0.02, 0.13)	0.04 (0.01, 0.12)
	≥ 1 STI year prior	19.13 (16.07, 22.77)	14.68 (11.43, 18.92)	8.66 (6.41, 11.69)	5.27 (3.1, 9.01)	0.21 (0.05, 0.84)	0.1 (0.01, 0.74)
	≥ 2 STI year prior	27.57 (20.58, 36.92)	19.74 (12.69, 31.09)	13.76 (7.81, 24.24)	6.4 (2.03, 20.48)	0.46 (0.06, 3.23)	0.46 (0.06, 3.23)
	≥ 3 STI year prior	45.29 (29.19, 70.21)	34.46 (18.26, 73.21)	21.52 (9.52, 48.56)	10.71 (2.12, 57.6)	0 (0, 0)	0 (0, 0)
Consistent PrEP[4]							
	All	8.45 (7.63, 9.37)	6.67 (5.77, 7.72)	4.09 (3.43, 4.88)	2.18 (1.58, 3.02)	0.07 (0.03, 0.19)	0.05 (0.02, 0.16)
	≥ 1 STI year prior	20.06 (16.73, 24.04)	15.86 (12.26, 20.59)	9.13 (6.6, 12.62)	5.83 (3.34, 10.25)	0.25 (0.06, 1.01)	0.13 (0.02, 0.89)
	≥ 2 STI year prior	30.11 (22.3, 40.62)	21.64 (13.82, 34.39)	13.71 (7.43, 25.26)	7.36 (2.33, 23.54)	0.52 (0.07, 3.71)	0.52 (0.07, 3.71)
	≥ 3 STI year prior	50.48 (32.78, 77.68)	38.41 (20.51, 81.07)	23.99 (10.6, 54.17)	11.94 (2.37, 64.12)	0 (0, 0)	0 (0, 0)
STI							
	≥ 1 STI year prior	5.96 (5.26, 6.76)	5.19 (4.36, 6.19)	5.09 (4.22, 6.14)	2.17 (1.57, 2.99)	0.07 (0.02, 0.17)	0.03 (0.01, 0.13)
	≥ 2 STI year prior	10.45 (8.29, 13.16)	9.46 (7, 12.85)	7.09 (5.18, 9.72)	3.64 (1.93, 6.96)	0.1 (0.01, 0.69)	0.1 (0.01, 0.69)
	≥ 3 STI year prior	21.3 (14.66, 30.91)	18.24 (11.3, 30.75)	8.5 (4.37, 16.54)	5.45 (1.76, 18.23)	0 (0, 0)	0 (0, 0)

CI: Confidence interval; STIs: Sexually-transmitted infections (gonorrhea, chlamydia, syphilis).

[1]People living with HIV (PLWH).

[2]PrEP use: filled ≥1 PrEP prescription in the previous year.

[3]Active PrEP use: ≥1 PrEP prescription in the previous three months.

[4]Consistent PrEP use: ≥1 PrEP prescription in the previous three months and ≥3 PrEP fills in the previous year.

Table S8: Incidence rate of oral and injection antibiotic fills related to sexually transmitted infections diagnoses among males and transgender individuals aged 35 to 44 years from 2017 to 2019 in the United States.

Cohort		Incidence of antibiotic fills related to STIs per 100-person years (95% CI)					
		Cephalosporins	*Macrolides*	*Penicillins*	*Tetracyclines*	*Quinolones*	*Aminoglycosides*
PLWH[1]							
	All	2.97 (2.45, 3.6)	2.34 (1.76, 3.14)	8.49 (7.2, 10.01)	1.27 (0.83, 1.95)	0.06 (0.02, 0.18)	0 (0, 0)
	≥ 1 STI year prior	12.07 (8.96, 16.24)	10.22 (6.49, 16.36)	24.96 (19.33, 32.22)	2.86 (1.26, 7.02)	0.2 (0.03, 1.43)	0 (0, 0)
	≥ 2 STI year prior	24.08 (15.78, 36.7)	21.76 (12.02, 40.6)	35.54 (22.37, 56.41)	2.28 (0.58, 9)	0 (0, 0)	0 (0, 0)
	≥ 3 STI year prior	33.21 (17.42, 63.22)	20.75 (9.81, 43.81)	41.47 (18.29, 93.91)	0 (0, 0)	0 (0, 0)	0 (0, 0)
PrEP[2]							
	All	5.21 (4.73, 5.74)	4.26 (3.7, 4.92)	2.56 (2.2, 2.98)	1.64 (1.21, 2.21)	0.08 (0.04, 0.18)	0.02 (0.01, 0.08)
	≥ 1 STI year prior	16.19 (13.42, 19.54)	11.28 (8.42, 15.23)	7.98 (6.08, 10.47)	5.58 (3.37, 9.27)	0 (0, 0)	0 (0, 0)
	≥ 2 STI year prior	27.87 (20.25, 38.32)	14.68 (8.51, 25.45)	13.67 (8.77, 21.29)	8.08 (3.53, 18.67)	0 (0, 0)	0 (0, 0)
	≥ 3 STI year prior	28.12 (16.02, 49.28)	15.29 (5.33, 45.5)	20.41 (8.6, 48.35)	12.72 (3.3, 57.89)	0 (0, 0)	0 (0, 0)
Active PrEP[3]							
	All	6.18 (5.59, 6.83)	4.97 (4.28, 5.78)	2.7 (2.29, 3.18)	1.94 (1.42, 2.63)	0.09 (0.04, 0.21)	0.03 (0.01, 0.1)
	≥ 1 STI year prior	17.46 (14.48, 21.04)	11.81 (8.65, 16.29)	8.66 (6.59, 11.37)	6.03 (3.52, 10.31)	0 (0, 0)	0 (0, 0)
	≥ 2 STI year prior	24.02 (17.39, 33.16)	14.28 (8.37, 24.8)	9.14 (5.26, 15.86)	10.27 (4.46, 24.21)	0 (0, 0)	0 (0, 0)
	≥ 3 STI year prior	22.02 (11.78, 41.11)	13.18 (4.35, 39.86)	8.79 (3.42, 22.59)	13.17 (4.09, 52.54)	0 (0, 0)	0 (0, 0)
Consistent PrEP[4]							
	All	6.43 (5.76, 7.17)	5.33 (4.55, 6.26)	2.9 (2.44, 3.45)	2.01 (1.43, 2.82)	0.11 (0.05, 0.26)	0.03 (0.01, 0.13)
	≥ 1 STI year prior	18.55 (15.31, 22.47)	12.46 (9.08, 17.28)	9.05 (6.87, 11.92)	5.92 (3.31, 10.61)	0 (0, 0)	0 (0, 0)
	≥ 2 STI year prior	25.45 (18.44, 35.09)	15.13 (8.87, 26.26)	9.08 (5.1, 16.13)	9.66 (4, 24.28)	0 (0, 0)	0 (0, 0)
	≥ 3 STI year prior	23.04 (12.36, 42.89)	13.79 (4.55, 41.65)	6.89 (2.28, 20.82)	9.18 (2.64, 36.32)	0 (0, 0)	0 (0, 0)
STI							
	≥ 1 STI year prior	7.57 (6.5, 8.81)	6.22 (5, 7.75)	8.55 (6.37, 11.48)	3.19 (2.3, 4.42)	0.05 (0.01, 0.21)	0.03 (0, 0.18)
	≥ 2 STI year prior	16.49 (12.57, 21.64)	12.95 (8.74, 19.19)	12.22 (9.18, 16.26)	5.59 (3.15, 9.93)	0 (0, 0)	0 (0, 0)
	≥ 3 STI year prior	31.78 (20.93, 48.22)	18.51 (10, 38.4)	22.94 (13.9, 37.82)	14.97 (6.53, 34.54)	0 (0, 0)	0 (0, 0)

CI: Confidence interval; STIs: Sexually-transmitted infections (gonorrhea, chlamydia, syphilis).
[1]People living with HIV (PLWH).
[2]PrEP use: filled ≥1 PrEP prescription in the previous year.
[3]Active PrEP use: ≥1 PrEP prescription in the previous three months.
[4]Consistent PrEP use: ≥1 PrEP prescription in the previous three months and ≥3 PrEP fills in the previous year.

Table S9: Incidence rate of oral and injection antibiotic fills related to sexually transmitted infections diagnoses among males and transgender individuals aged 45 to 65 years from 2017 to 2019 in the United States.

Cohort		Incidence of antibiotic fills related to STIs per 100-person years (95% CI)					
		Cephalosporins	*Macrolides*	*Penicillins*	*Tetracyclines*	*Quinolones*	*Aminoglycosides*
PLWH[1]							
	All	1.39 (1.21, 1.6)	0.96 (0.78, 1.18)	4.6 (4.1, 5.16)	0.8 (0.61, 1.06)	0.02 (0, 0.05)	0.01 (0, 0.04)
	≥ 1 STI year prior	10 (7.83, 12.76)	6.58 (4.68, 9.5)	21.88 (17.37, 27.54)	4.58 (2.55, 8.35)	0 (0, 0)	0 (0, 0)
	≥ 2 STI year prior	21.05 (14.05, 31.5)	11.68 (6.29, 24.67)	21.02 (11.39, 38.75)	10.88 (4.77, 27.71)	0 (0, 0)	0 (0, 0)
	≥ 3 STI year prior	32.39 (17.43, 60.1)	14.9 (4.96, 50.65)	29.83 (10.81, 82.17)	9.92 (2.56, 38.37)	0 (0, 0)	0 (0, 0)
PrEP[2]							
	All	2.69 (2.34, 3.08)	2.17 (1.85, 2.55)	3.73 (3.17, 4.39)	0.98 (0.73, 1.3)	0.05 (0.02, 0.1)	0.01 (0, 0.05)
	≥ 1 STI year prior	15.23 (11.2, 20.7)	11.02 (8.27, 14.77)	12.32 (9.15, 16.58)	2.48 (1.25, 4.99)	0.21 (0.05, 0.86)	0 (0, 0)
	≥ 2 STI year prior	28.39 (15.2, 52.96)	15.04 (8.63, 26.6)	8.9 (4.52, 17.52)	6.67 (2.82, 17.54)	0.55 (0.08, 3.83)	0 (0, 0)
	≥ 3 STI year prior	46.02 (13.22, 159.83)	16.95 (4.8, 60.9)	0 (0, 0)	12.12 (3.86, 38.65)	2.41 (0.36, 16)	0 (0, 0)
Active PrEP[3]							
	All	3.1 (2.68, 3.58)	2.57 (2.18, 3.05)	3.56 (2.96, 4.29)	1.08 (0.79, 1.49)	0.06 (0.03, 0.12)	0.02 (0, 0.06)
	≥ 1 STI year prior	15.41 (10.79, 21.98)	10.63 (7.61, 14.86)	11.43 (8.57, 15.24)	3.05 (1.48, 6.39)	0.53 (0.2, 1.41)	0 (0, 0)
	≥ 2 STI year prior	26.13 (12.57, 54.25)	15.31 (8.34, 28.21)	7.65 (4.05, 14.43)	4.45 (1.62, 12.33)	0.63 (0.09, 4.36)	0 (0, 0)
	≥ 3 STI year prior	53.74 (14.69, 196.05)	29.93 (12.69, 74.42)	2.97 (0.42, 21.05)	8.95 (2.01, 42.91)	0 (0, 0)	0 (0, 0)
Consistent PrEP[4]							
	All	3.11 (2.69, 3.59)	2.61 (2.17, 3.14)	3.93 (3.23, 4.78)	1.14 (0.81, 1.6)	0.05 (0.02, 0.11)	0.02 (0, 0.08)
	≥ 1 STI year prior	14.15 (10.22, 19.58)	11.62 (8.31, 16.28)	11.32 (8.34, 15.37)	3.27 (1.56, 7.01)	0.44 (0.14, 1.38)	0 (0, 0)
	≥ 2 STI year prior	22.68 (10.44, 49.19)	16.31 (8.86, 30.32)	6.38 (3.15, 12.92)	4.95 (1.8, 13.74)	0.7 (0.1, 4.83)	0 (0, 0)
	≥ 3 STI year prior	57.18 (15.67, 208.08)	31.84 (13.54, 79.17)	0 (0, 0)	9.52 (2.15, 45.54)	0 (0, 0)	0 (0, 0)
STI							
	≥ 1 STI year prior	6.46 (5.39, 7.73)	5.29 (4.31, 6.53)	10.48 (8.95, 12.26)	2.24 (1.57, 3.21)	0.11 (0.05, 0.26)	0 (0, 0)
	≥ 2 STI year prior	14.67 (10.21, 21.07)	10.6 (7.03, 16.24)	9.3 (6.5, 13.29)	5.37 (3.1, 9.44)	0.14 (0.02, 1.02)	0 (0, 0)
	≥ 3 STI year prior	28.78 (14.49, 57.06)	19.17 (8.21, 45.27)	11.06 (4.66, 26.17)	10.32 (4.42, 25.78)	0.73 (0.1, 5.11)	0 (0, 0)

CI: Confidence interval; STIs: Sexually-transmitted infections (gonorrhea, chlamydia, syphilis).
[1]People living with HIV (PLWH).
[2]PrEP use: filled ≥1 PrEP prescription in the previous year.
[3]Active PrEP use: ≥1 PrEP prescription in the previous three months.
[4]Consistent PrEP use: ≥1 PrEP prescription in the previous three months and ≥3 PrEP fills in the previous year.

Table S10: Incidence rate of oral and injection antibiotic fills related to gonorrhea diagnoses among males and transgender individuals from 2017 to 2019 in the United States.

Cohort		Incidence rate of antibiotic fills related to gonorrhea diagnoses per 100-person years (95% CI)				
		Cephalosporins	*Macrolides*	*Tetracyclines*	*Quinolones*	*Aminoglycosides*
PLWH[1]						
	All	2.21 (2.01, 2.43)	1.3 (1.15, 1.46)	0.23 (0.17, 0.3)	0.04 (0.02, 0.07)	0.01 (0, 0.03)
	≥1 STI in year prior	10.8 (9.25, 12.62)	6.41 (5.21, 7.88)	0.93 (0.57, 1.51)	0.1 (0.03, 0.41)	0 (0, 0)
	≥2 STIs in year prior	19.26 (14.93, 24.83)	10.72 (7.6, 15.13)	1.09 (0.33, 3.61)	0 (0, 0)	0 (0, 0)
	≥3 STIs in year prior	31.51 (21.74, 45.65)	14.69 (8.74, 24.68)	0 (0, 0)	0 (0, 0)	0 (0, 0)
PrEP use[2]						
	All	4.48 (4.23, 4.76)	2.86 (2.67, 3.05)	0.40 (0.33, 0.47)	0.06 (0.04, 0.1)	0.03 (0.01, 0.05)
	≥1 STI in year prior	15.92 (14.06, 18.03)	8.92 (7.78, 10.21)	1.34 (0.93, 1.92)	0.13 (0.05, 0.34)	0.03 (0, 0.22)
	≥2 STIs in year prior	25 (19.67, 31.76)	10.82 (8.41, 13.91)	2.63 (1.43, 4.82)	0.14 (0.02, 1.02)	0 (0, 0)
	≥3 STIs in year prior	35.66 (21.75, 58.42)	11.89 (7.19, 19.64)	4.61 (2.03, 10.45)	0.66 (0.09, 4.57)	0 (0, 0)
Active PrEP use[3]						
	All	5.27 (4.95, 5.6)	3.42 (3.18, 3.67)	0.45 (0.38, 0.55)	0.07 (0.04, 0.11)	0.03 (0.01, 0.05)
	≥1 STI in year prior	17.29 (15.21, 19.65)	9.64 (8.37, 11.1)	1.69 (1.2, 2.38)	0.22 (0.1, 0.5)	0.04 (0.01, 0.26)
	≥2 STIs in year prior	25.33 (19.72, 32.52)	12.58 (9.73, 16.26)	2.68 (1.38, 5.2)	0.33 (0.08, 1.33)	0.17 (0.02, 1.18)
	≥3 STIs in year prior	36.46 (21.6, 61.45)	19.7 (12.74, 30.42)	4.37 (1.76, 10.82)	0 (0, 0)	0 (0, 0)
Consistent PrEP use[4]						
	All	5.48 (5.14, 5.86)	3.64 (3.37, 3.94)	0.49 (0.4, 0.6)	0.08 (0.05, 0.13)	0.03 (0.01, 0.06)
	≥1 STI in year prior	17.65 (15.58, 19.98)	10.48 (9.05, 12.13)	1.72 (1.18, 2.51)	0.22 (0.09, 0.53)	0.04 (0.01, 0.31)
	≥2 STIs in year prior	26.06 (20.45, 33.2)	13.79 (10.63, 17.87)	3.01 (1.55, 5.85)	0.38 (0.09, 1.49)	0.19 (0.03, 1.33)
	≥3 STIs in year prior	39.8 (23.64, 66.95)	21.5 (13.95, 33.13)	4.77 (1.92, 11.79)	0 (0, 0)	0 (0, 0)
STI history						
	≥1 STI in year prior	5.46 (5.04, 5.93)	3.43 (3.13, 3.76)	0.59 (0.48, 0.72)	0.07 (0.04, 0.11)	0.06 (0.02, 0.23)
	≥2 STIs in year prior	11.32 (9.67, 13.25)	6.9 (5.79, 8.21)	1.35 (0.94, 1.93)	0.06 (0.02, 0.25)	0.03 (0, 0.23)
	≥3 STIs in year prior	24.2 (18.18, 32.18)	11.39 (8.5, 15.25)	2.44 (1.33, 4.45)	0.2 (0.03, 1.42)	0 (0, 0)

CI: Confidence interval; STIs: Sexually-transmitted infections (gonorrhea, chlamydia, syphilis).
[1]People living with HIV (PLWH).
[2]PrEP use: filled ≥1 PrEP prescription in the previous year.
[3]Active PrEP use: ≥1 PrEP prescription in the previous three months.
[4]Consistent PrEP use: ≥1 PrEP prescription in the previous three months and ≥3 PrEP fills in the previous year.

Table S11: Incidence rate of oral and injection antibiotic fills related to chlamydia diagnoses with and without a gonorrhea co-infection from 2017 to 2019 in the United States.

Cohort		Incidence rate of antibiotic fills related to STIs per 100-person years (95% CI)			
		All chlamydia cases		*Chlamydia cases without gonorrhea co-infection*	
		Macrolides	Tetracyclines	Macrolides	Tetracyclines
PLWH[1]					
	All	0.48 (0.39, 0.58)	0.29 (0.23, 0.37)	0.3 (0.24, 0.37)	0.24 (0.18, 0.31)
	≥1 STI in year prior	2.48 (1.69, 3.63)	1.24 (0.82, 1.87)	1.34 (0.86, 2.1)	0.93 (0.57, 1.51)
	≥2 STIs in year prior	3.57 (2.02, 6.31)	3.02 (1.68, 5.41)	1.92 (0.92, 4)	2.47 (1.29, 4.72)
	≥3 STIs in year prior	4.19 (1.62, 10.83)	2.09 (0.53, 8.25)	1.04 (0.15, 7.33)	2.09 (0.53, 8.25)
PrEP use[2]					
	All	0.94 (0.84, 1.05)	0.56 (0.49, 0.65)	0.69 (0.6, 0.78)	0.45 (0.38, 0.53)
	≥1 STI in year prior	2.77 (2.22, 3.45)	1.65 (1.25, 2.19)	2.23 (1.75, 2.83)	1.3 (0.96, 1.78)
	≥2 STIs in year prior	4.24 (2.93, 6.12)	2.34 (1.44, 3.78)	3.51 (2.33, 5.28)	1.46 (0.79, 2.7)
	≥3 STIs in year prior	5.28 (2.46, 11.28)	3.96 (1.81, 8.63)	4.61 (2.02, 10.54)	1.97 (0.64, 6.06)
Active PrEP use[3]					
	All	1.11 (0.99, 1.25)	0.66 (0.57, 0.77)	0.83 (0.73, 0.95)	0.54 (0.46, 0.64)
	≥1 STI in year prior	3.71 (3.03, 4.55)	2.25 (1.72, 2.95)	2.92 (2.32, 3.68)	1.84 (1.35, 2.49)
	≥2 STIs in year prior	5.2 (3.68, 7.34)	3.35 (2.04, 5.48)	3.86 (2.58, 5.76)	2.68 (1.51, 4.74)
	≥3 STIs in year prior	6.56 (3.47, 12.36)	5.1 (2.46, 10.54)	5.1 (2.47, 10.51)	3.64 (1.53, 8.64)
Consistent PrEP use[4]					
	All	1.11 (0.97, 1.26)	0.69 (0.58, 0.82)	0.82 (0.7, 0.95)	0.55 (0.45, 0.67)
	≥1 STI in year prior	3.58 (2.87, 4.47)	2.3 (1.71, 3.08)	2.83 (2.21, 3.62)	1.86 (1.33, 2.59)
	≥2 STIs in year prior	5.1 (3.52, 7.37)	3.39 (2, 5.75)	3.77 (2.45, 5.8)	2.64 (1.42, 4.91)
	≥3 STIs in year prior	7.16 (3.79, 13.49)	4.77 (2.17, 10.48)	5.57 (2.7, 11.47)	3.18 (1.2, 8.38)
STI history					
	≥1 STI in year prior	2.03 (1.81, 2.29)	0.96 (0.83, 1.11)	1.46 (1.28, 1.67)	0.76 (0.64, 0.89)
	≥2 STIs in year prior	4.94 (4.07, 6)	1.76 (1.36, 2.29)	3.27 (2.57, 4.17)	1.19 (0.86, 1.63)
	≥3 STIs in year prior	8.33 (5.3, 13.08)	2.84 (1.7, 4.75)	5.48 (2.94, 10.2)	1.62 (0.82, 3.23)

CI: Confidence interval; STIs: Sexually-transmitted infections (gonorrhea, chlamydia, syphilis).
[1]People living with HIV (PLWH).
[2]PrEP use: filled ≥1 PrEP prescription in the previous year.
[3]Active PrEP use: ≥1 PrEP prescription in the previous three months.
[4]Consistent PrEP use: ≥1 PrEP prescription in the previous three months and ≥3 PrEP fills in the previous year.

Table S12: Incidence rate of oral and injection antibiotic fills related to syphilis diagnoses from 2017 to 2019 in the United States.

Cohort		Incidence rate of antibiotic fills related to syphilis diagnoses per 100-person years (95% CI)	
		Penicillins	*Tetracyclines*
PLWH[1]			
	All	6.17 (5.7, 6.68)	0.66 (0.57, 0.77)
	≥1 STI in year prior	21.81 (18.87, 25.21)	2.48 (1.8, 3.42)
	≥2 STIs in year prior	23.1 (17.1, 31.19)	3.57 (2.01, 6.33)
	≥3 STIs in year prior	29.38 (16.8, 51.32)	2.09 (0.53, 8.25)
PrEP use[2]			
	All	3.33 (3.03, 3.66)	0.55 (0.47, 0.63)
	≥1 STI in year prior	9.42 (7.97, 11.13)	1.46 (1.1, 1.95)
	≥2 STIs in year prior	10.23 (7.54, 13.87)	1.9 (1.11, 3.25)
	≥3 STIs in year prior	10.56 (5.77, 19.3)	2.63 (1, 6.89)
Active PrEP use[3]			
	All	3.3 (2.98, 3.66)	0.57 (0.49, 0.67)
	≥1 STI in year prior	9.38 (7.98, 11.02)	1.35 (0.97, 1.87)
	≥2 STIs in year prior	10.4 (7.41, 14.58)	1.51 (0.79, 2.88)
	≥3 STIs in year prior	11.66 (6.45, 21.06)	2.18 (0.71, 6.7)
Consistent PrEP use[4]			
	All	3.64 (3.25, 4.08)	0.62 (0.52, 0.74)
	≥1 STI in year prior	9.42 (7.91, 11.21)	1.46 (1.04, 2.05)
	≥2 STIs in year prior	9.62 (6.59, 14.05)	1.51 (0.75, 3.01)
	≥3 STIs in year prior	10.34 (5.21, 20.49)	1.58 (0.4, 6.31)
STI history			
	≥1 STI in year prior	5.96 (5.3, 6.69)	0.78 (0.66, 0.93)
	≥2 STIs in year prior	7.31 (6.12, 8.74)	1.44 (1.06, 1.96)
	≥3 STIs in year prior	11.58 (8.02, 16.72)	4.06 (2.54, 6.5)

CI: Confidence interval; STIs: Sexually-transmitted infections (gonorrhea, chlamydia, syphilis).
[1]People living with HIV (PLWH).
[2]PrEP use: filled ≥1 PrEP prescription in the previous year.
[3]Active PrEP use: ≥1 PrEP prescription in the previous three months.
[4]Consistent PrEP use: ≥1 PrEP prescription in the previous three months and ≥3 PrEP fills in the previous year.

Table S13: Incidence rate difference of sexually transmitted infections among males and transgender individuals with 100% doxycycline postexposure prophylaxis (doxyPEP) uptake.

Cohort		Incidence rate difference per 100 person-years (95% CI)		
		Gonorrhea	*Chlamydia*	*Syphilis*
PLWH[1]				
	All	-1.5 (-2.2, -0.4)	-1.5 (-1.8, -1.0)	-2.3 (-2.6, -1.9)
	≥1 STI in year prior	-7.4 (-11.1, -1.8)	-7.3 (-9.4, -4.9)	-5.9 (-7.2, -4.6)
	≥2 STIs in year prior	-14.3 (-22.4, -3.6)	-14.9 (-20.4, -9.5)	-8.0 (-11.1, -5.7)
	≥3 STIs in year prior	-22.3 (-37.8, -5.6)	-23.3 (-38.9, -12.8)	-10.4 (-17.5, -6.1)
PrEP use[2]				
	All	-2.7 (-3.9, -0.7)	-2.5 (-2.9, -1.7)	-1.4 (-1.6, -1.1)
	≥1 STI in year prior	-9.6 (-14.3, -2.4)	-8.1 (-9.8, -5.4)	-3.1 (-3.9, -2.4)
	≥2 STIs in year prior	-16.7 (-25.5, -4.1)	-14.4 (-18.8, -9.5)	-3.0 (-4.5, -2.0)
	≥3 STIs in year prior	-22.0 (-36.6, -5.4)	-23.1 (-34.8, -13.9)	-3.0 (-6.7, -1.4)
Active PrEP use[3]				
	All	-3.1 (-4.6, -0.8)	-2.9 (-3.3, -1.9)	-1.5 (-1.7, -1.2)
	≥1 STI in year prior	-11.2 (-16.6, -2.7)	-10.8 (-13.1, -7.3)	-2.8 (-3.6, -2.1)
	≥2 STIs in year prior	-18.1 (-27.6, -4.6)	-18.3 (-23.7, -12)	-3.3 (-5.0, -2.2)
	≥3 STIs in year prior	-27.7 (-44.6, -6.8)	-30.8 (-44.7, -19)	-3.3 (-7.6, -1.5)
Consistent PrEP use[4]				
	All	-3.3 (-4.8, -0.8)	-3.0 (-3.5, -2.0)	-1.6 (-1.8, -1.3)
	≥1 STI in year prior	-11.5 (-17.2, -2.8)	-11.0 (-13.3, -7.4)	-3.0 (-3.8, -2.3)
	≥2 STIs in year prior	-18.9 (-28.8, -4.7)	-18.9 (-24.7, -12.4)	-3.2 (-4.8, -2.0)
	≥3 STIs in year prior	-28.8 (-46.6, -7.1)	-31.7 (-47.1, -19.5)	-2.4 (-6.6, -0.9)
STI history				
	≥1 STI in year prior	-3.8 (-5.7, -1.0)	-5.8 (-6.7, -3.9)	-1.6 (-1.9, -1.3)
	≥2 STIs in year prior	-8.9 (-13.4, -2.2)	-13.2 (-16.0, -9.0)	-2.5 (-3.1, -1.9)
	≥3 STIs in year prior	-17.9 (-27.6, -4.4)	-29.2 (-39.1, -18.8)	-4.5 (-6.6, -3.0)

CI: Confidence interval; STIs: Sexually-transmitted infections (gonorrhea, chlamydia, syphilis).
[1]People living with HIV (PLWH).
[2]PrEP use: filled ≥1 PrEP prescription in the previous year.
[3]Active PrEP use: ≥1 PrEP prescription in the previous three months.
[4]Consistent PrEP use: ≥1 PrEP prescription in the previous three months and ≥3 PrEP fills in the previous year.

Table S14: Estimates of incidence rate difference in standardized tetracycline fills with 100% doxycycline postexposure prophylaxis (doxyPEP) uptake utilizing alternative estimates of sexual encounter frequencies.

Cohort		Incidence rate difference for tetracycline fill-days related to STIs per 100 person-years (95% CI)	
		Reference manuscript	
		Grov et al., 2016	Janulis et al., 2023
PLWH			
	All	141.0 (140.9, 141.2)	142.1 (142.0, 142.4)
	≥1 STI year prior	188.0 (187.0, 188.9)	2401.8 (2401.2, 2403.1)
	≥2 STI year prior	319.1 (315.3, 321.6)	2833.2 (2832.0, 2837.0)
	≥3 STI year prior	659.1 (649.5, 661.5)	3242.6 (3241.9, 3245.6)
PrEP use			
	All	203.5 (203.5, 203.7)	204.9 (204.8, 205.2)
	≥1 STI year prior	251.1 (250.6, 251.7)	2147.7 (2147.1, 2148.9)
	≥2 STI year prior	359.2 (357.7, 360.6)	2452.2 (2450.9, 2455.7)
	≥3 STI year prior	578.4 (572.8, 581.4)	2761.1 (2758.5, 2769.7)
STI history			
	≥1 STI year prior	113.7 (113.5, 113.9)	2275.9 (2275.7, 2276.4)
	≥2 STI year prior	169.7 (169.2, 170.3)	2714.2 (2713.6, 2715.4)
	≥3 STI year prior	316.9 (314.3, 319.0)	2753.8 (2752.3, 2757.5)

CI: Confidence interval; STIs: Sexually-transmitted infections (gonorrhea, chlamydia, syphilis).
Incidence rates and incidence rate differences are reported per 100 person-years. We equated a 200 mg doxycycline dose to one-seventh of standardized tetracycline fill, reflecting the standard seven-day treatment regimen for chlamydia. The incidence rate difference in standardized tetracycline fills is equal to the increase in standardized tetracycline fills for those on doxyPEP, less STI-related tetracycline fills prevented by doxyPEP. Due to limited empirical estimates among transgender individuals, estimates are derived from men who have sex with men studies[1,10–13]. See **Table S12** for the estimated annual rates of new or casual unprotected anal sex partnerships utilizing each reference manuscript. All other tables use data from the Jin et al. study to estimate doxycycline use with doxyPEP implementation.

Table S15. Incidence rate difference of oral and injection antibiotic fills related to sexually transmitted infections among males and transgender individuals with doxycycline postexposure prophylaxis having no effect on gonorrhea infections.

Cohort		Incidence rate difference of antibiotic fills related to STIs per 100 person-years (95% CI)			
		Cephalosporins	*Macrolides*	*Penicillins*	*Tetracyclines*
PLWH[1]					
	All	0 (0, 0)	-0.2 (-0.2, -0.2)	-4.8 (-5.5, -3.8)	141.0 (140.9, 141.2)
	≥1 STI in year prior	0 (0, 0)	-1.1 (-1.2, -0.8)	-16.7 (-20.2, -13.1)	271.9 (270.9, 272.8)
	≥2 STIs in year prior	0 (0, 0)	-1.6 (-2.2, -0.9)	-17.7 (-24.4, -12.4)	461.1 (457.3, 463.6)
	≥3 STIs in year prior	0 (0, 0)	-0.8 (-4.0, -0.1)	-22.4 (-39.6, -12.5)	647.7 (638.1, 650.1)
PrEP use[2]					
	All	0 (0, 0)	-0.6 (-0.6, -0.4)	-2.6 (-.03, -2.1)	203.5 (203.4, 203.7)
	≥1 STI in year prior	0 (0, 0)	-1.8 (-1.8, -1.5)	-7.2 (-8.9, -5.6)	312.9 (312.4, 313.5)
	≥2 STIs in year prior	0 (0, 0)	-2.8 (-3.1, -2.1)	-7.8 (-10.9, -5.5)	421.4 (419.9, 422.8)
	≥3 STIs in year prior	0 (0, 0)	-3.7 (-5.8, -1.8)	-8.1 (-14.8, -4.3)	532.4 (526.8, 535.4)
Active PrEP use[3]					
	All	0 (0, 0)	-0.7 (-0.7, -0.5)	-2.5 (-3.0, -2.0)	203.4 (203.3, 203.6)
	≥1 STI in year prior	0 (0, 0)	-2.3 (-2.4, -2.0)	-7.2 (-8.8, -5.6)	312.4 (311.8, 313.2)
	≥2 STIs in year prior	0 (0, 0)	-3.1 (-3.3, -2.4)	-7.9 (-11.4, -5.4)	420.7 (418.5, 422.5)
	≥3 STIs in year prior	0 (0, 0)	-4.1 (-5.7, -2.3)	-8.9 (-16.2, -4.8)	531.5 (524.8, 535.1)
Consistent PrEP use[4]					
	All	0 (0, 0)	-0.7 (-0.7, -0.5)	-2.8 (-3.3, -2.2)	203.4 (203.2, 203.5)
	≥1 STI in year prior	0 (0, 0)	-2.3 (-2.3, -1.9)	-7.2 (-8.9, -5.6)	312.3 (311.6, 313.1)
	≥2 STIs in year prior	0 (0, 0)	-3.1 (-3.3, -2.3)	-7.4 (-10.9, -4.8)	420.6 (418.2, 422.5)
	≥3 STIs in year prior	0 (0, 0)	-4.5 (-6.3, -2.5)	-7.9 (-15.7, -3.9)	532.2 (525., 535.50)
STI history					
	≥1 STI in year prior	0 (0, 0)	-1.2 (-1.2, -0.9)	-4.6 (-5.4, -3.6)	174.6 (174.5, 174.9)
	≥2 STIs in year prior	0 (0, 0)	-2.6 (-2.7, -2.2)	-5.6 (-6.9, -4.3)	299.2 (298.7, 299.7)
	≥3 STIs in year prior	0 (0, 0)	-4.4 (-5.6, -2.7)	-8.9 (-13.0, -5.9)	447.2 (444.6, 449.2)

CI: Confidence interval; STIs: Sexually-transmitted infections (gonorrhea, chlamydia, syphilis).
[1]People living with HIV (PLWH).
[2]PrEP use: filled ≥1 PrEP prescription in the previous year.
[3]Active PrEP use: ≥1 PrEP prescription in the previous three months.
[4]Consistent PrEP use: ≥1 PrEP prescription in the previous three months and ≥3 PrEP fills in the previous year.

Table S16: Estimated increase in standardized tetracycline fills per sexually transmitted infection prevented.

DoxyPEP eligible population	Cohort	Excess standardized tetracycline fill-days per outcome prevented (95% CI)		
		Gonorrhea	*Chlamydia*	*Syphilis*
Entire cohort				
	PLWH	93.9 (69.2, 292.1)	95.7 (80.4, 141.9)	61.0 (53.5, 75.8)
	PrEP use	76.0 (54.3, 243.2)	82.5 (71.1, 121.6)	145.3 (126.9, 180.3)
	Active PrEP use	64.8 (46.4, 206.8)	71.2 (61.2, 105.0)	139.9 (121.6, 173.8)
	Consistent PrEP use	61.7 (44.4, 195.8)	69.0 (58.9, 101.9)	128.8 (111.3, 160.3)
≥ 1 STI in year prior				
	PLWH	36.5 (28.4, 108.0)	37.0 (29.0, 56.2)	46.1 (37.6, 59.3)
	PrEP use	32.2 (24.1, 98.8)	38.7 (31.8, 57.9)	100.2 (81.4, 129.3)
	Active PrEP use	27.8 (20.8, 85.4)	28.9 (23.9, 43.0)	109.9 (87.5, 144.0)
	Consistent PrEP use	26.9 (20.2, 81.9)	28.4 (23.3, 42.4)	103.7 (81.9, 136.8)
	STI History	45.2 (32.7, 142.9)	30.2 (26.1, 44.6)	108.5 (93.4, 135.7)
≥ 2 STIs in year prior				
	PLWH	31.9 (27.0, 87.1)	30.9 (22.3, 48.7)	57.8 (41.6, 82.3)
	PrEP use	25.0 (20.1, 72.0)	29.2 (22.3, 44.9)	139.9 (94.7, 211.2)
	Active PrEP use	22.9 (18.3, 66.3)	23.0 (17.7, 35.2)	126.4 (84.8, 192.4)
	Consistent PrEP use	22.0 (17.7, 63.3)	22.2 (17.0, 34.1)	132.7 (86.2, 208.2)
	STI History	33.2 (25.2, 100.5)	22.6 (18.7, 33.6)	121.8 (97.0, 159.8)
≥ 3 STIs in year prior				
	PLWH	28.4 (26.2, 70.1)	27.8 (16.6, 49.7)	62.2 (36.8, 106.3)
	PrEP use	23.7 (21.6, 60.1)	23.0 (15.2, 38.3)	176.1 (79.7, 392.9)
	Active PrEP use	18.9 (16.7, 49.5)	17.3 (11.8, 28.1)	159.2 (70.9, 360.6)
	Consistent PrEP use	18.2 (16.2, 47.3)	16.8 (11.3, 27.4)	219.0 (82.0, 593.2)
	STI History	24.8 (20.2, 69.9)	15.3 (11.4, 23.6)	99.3 (68.1, 148.0)

CI: Confidence interval; STIs: Sexually-transmitted infections (gonorrhea, chlamydia, syphilis).
Estimates can be interpreted as the cohort-wide increase in tetracycline fill-days per incident STI case prevented, with doxyPEP targeted to the indicated eligible population.
[1]People living with HIV (PLWH).
[2]PrEP use: filled ≥1 PrEP prescription in the previous year.
[3]Active PrEP use: ≥1 PrEP prescription in the previous three months.
[4]Consistent PrEP use: ≥1 PrEP prescription in the previous three months and ≥3 PrEP fills in the previous year.

Table S17: Codes used to identify transgender persons.

Code type	Code	Code description
ICD10	F64	Gender identity disorders
ICD10	F64.0	Transsexualism
ICD10	F64.1	Gender identity disorder in adolescence and adulthood
ICD10	F64.2	Gender identity disorder in childhood
ICD10	F64.8	Other gender identity disorders
ICD10	F64.9	Gender identity disorder, unspecified
ICD10	Z87.890	Personal history of sex reassignment
ICD10-PCS	0W4M070	Creation of Vagina in Male Perineum with Autologous Tissue Substitute, Open Approach.
ICD10-PCS	0W4M0J0	Creation of Vagina in Male Perineum with Synthetic Substitute, Open Approach
ICD10-PCS	0W4M0K0	Creation of Vagina in Male Perineum with Nonautologous Tissue Substitute, Open Approach
ICD10-PCS	0W4M0Z0	Creation of Vagina in Male Perineum, Open Approach
ICD10-PCS	0W4N071	Creation of Penis in Female Perineum with Autologous Tissue Substitute, Open Approach
ICD10-PCS	0W4N0J1	Creation of Penis in Female Perineum with Synthetic Substitute, Open Approach
ICD10-PCS	0W4N0K1	Creation of Penis in Female Perineum with Nonautologous Tissue Substitute, Open Approach
ICD10-PCS	0W4N0Z1	Creation of Penis in Female Perineum, Open Approach

Table S18: National Drug Codes used to identify Emtricitabine/Tenofovir use.

Code type	Code	Code description
NDC	61958-0701-1	Truvada [emtricitabine/ tenofovir disoproxil fumarate], 200 mg/1, 300 mg/1
NDC	61958-0703-1	Truvada [emtricitabine/ tenofovir disoproxil fumarate], 100 mg/1, 150 mg/1
NDC	61958-0704-1	Truvada [emtricitabine/ tenofovir disoproxil fumarate], 133 mg/1, 200 mg/1
NDC	61958-0705-1	Truvada [emtricitabine/ tenofovir disoproxil fumarate], 167 mg/1, 250 mg/1
NDC	61958-2005-1	Descovy [emtricitabine and tenofovir alafenamide] 120 mg/1, 15 mg/1
NDC	61958-2002-2	Descovy [emtricitabine and tenofovir alafenamide] 200 mg/1, 25 mg/1
NDC	61958-2002-1	Descovy [emtricitabine and tenofovir alafenamide] 200 mg/1, 25 mg/1
NDC	70771-1620-2	Emtricitabine / Tenofovir
NDC	70771-1620-3	Emtricitabine / Tenofovir
NDC	70771-1620-4	Emtricitabine / Tenofovir
NDC	70771-1620-9	Emtricitabine / Tenofovir
NDC	70771-1621-2	Emtricitabine / Tenofovir
NDC	70771-1621-3	Emtricitabine / Tenofovir
NDC	70771-1621-4	Emtricitabine / Tenofovir
NDC	70771-1621-9	Emtricitabine / Tenofovir
NDC	70771-1622-2	Emtricitabine / Tenofovir
NDC	70771-1622-3	Emtricitabine / Tenofovir
NDC	70771-1622-4	Emtricitabine / Tenofovir
NDC	70771-1622-9	Emtricitabine / Tenofovir
NDC	70771-1709-2	Emtricitabine / Tenofovir
NDC	70771-1709-3	Emtricitabine / Tenofovir
NDC	70771-1709-4	Emtricitabine / Tenofovir
NDC	70771-1709-9	Emtricitabine / Tenofovir

Table S19: Codes used to identify HIV infection.

Code type	Code	Code description
ICD10	B20.0	HIV disease resulting in mycobacterial infection
ICD10	B20.1	HIV disease resulting in other bacterial infections
ICD10	B20.2	HIV disease resulting in cytomegaloviral disease
ICD10	B20.3	HIV disease resulting in other viral infections
ICD10	B20.4	HIV disease resulting in candidiasis
ICD10	B20.5	HIV disease resulting in other mycoses
ICD10	B20.6	HIV disease resulting in Pneumocystis jirovecii pneumonia
ICD10	B20.7	HIV disease resulting in multiple infections
ICD10	B20.8	HIV disease resulting in other infectious and parasitic diseases
ICD10	B20.9	HIV disease resulting in unspecified infectious or parasitic disease
ICD10	B21.0	HIV disease resulting in Kaposi sarcoma
ICD10	B21.1	HIV disease resulting in Burkitt lymphoma
ICD10	B21.2	HIV disease resulting in other types of non-Hodgkin lymphoma
ICD10	B21.3	HIV disease resulting in other malignant neoplasms of lymphoid, haematopoietic and related tissue
ICD10	B21.7	HIV disease resulting in multiple malignant neoplasms
ICD10	B21.8	HIV disease resulting in other malignant neoplasms
ICD10	B21.9	HIV disease resulting in unspecified malignant neoplasm
ICD10	B22.0	HIV disease resulting in encephalopathy HIV dementia
ICD10	B22.1	HIV disease resulting in lymphoid interstitial pneumonitis
ICD10	B22.2	HIV disease resulting in wasting syndrome
ICD10	B22.7	HIV disease resulting in multiple diseases classified elsewhere
ICD10	B23.0	Acute HIV infection syndrome
ICD10	B23.1	HIV disease resulting in (persistent) generalized lymphadenopathy
ICD10	B23.2	HIV disease resulting in haematological and immunological abnormalities, not elsewhere classified
ICD10	B23.8	HIV disease resulting in other specified conditions
ICD10	B24	Unspecified human immunodeficiency virus [HIV] disease
ICD10	O98.7	HIV complicating pregnancy, childbirth, or the puerperium
ICD10	O98.71X	HIV complicating pregnancy
ICD10	O98.72	HIV complicating childbirth
ICD10	O98.73	HIV complicating puerperium
ICD10	Z21	Asymptomatic HIV infection

Table S20: Codes used to identify HIV-associated opportunistic infections.

Code type	Code	Code description
ICD10	A07.2	Cryptosporidiosis
ICD10	A31.0	Pulmonary mycobacterial infection
ICD10	A31.2	Disseminated mycobacterium avium
ICD10	B25.X	Cytomegaloviral disease
ICD10	B37.1	Pulmonary candidiasis
ICD10	B37.81	Candidal esophagitis
ICD10	B38.X	Coccidioidomycosis
ICD10	B45.X	Cryptococcosis
ICD10	B58.2	Toxoplasma meningoencephalitis
ICD10	B59	Pneumocystosis
ICD10	C46.X	Kaposi's sarcoma

Table S21: Codes used to identify Hepatitis B infection.

Code type	Code	Code description
ICD10	B16x	Acute Hep B
ICD10	B16.0	Acute hepatitis B with delta-agent with hepatic coma
ICD10	B16.1	Acute hepatitis B with delta-agent without hepatic coma
ICD10	B16.2	Acute hepatitis B without delta-agent with hepatic coma
ICD10	B16.9	Acute hepatitis B without delta-agent and without hepatic coma
ICD10	B17.0	Acute hepatitis delta infection
ICD10	B17.8	Other specified acute viral hepatitis
ICD10	B18.0	Chronic hepatitis B with delta agent
ICD10	B18.1	Chronic hepatitis B no delta agent
ICD10	B19.1	Unspecified viral hepatitis B
ICD10	B19.10	Hepatitis B without hepatic coma
ICD10	B19.11	Hepatitis B with hepatic coma
ICD10	O98.419	Viral hepatitis complicating pregnancy, unspecified trimester
ICD10	Z20.5	Contact with and (suspected) exposure to viral hepatitis
ICD10	Z11.59	Encounter for screening for other viral diseases
ICD10	Z22.51	Hepatitis B carrier

Table S22: Codes used to identify needlestick exposure.

Code type	Code	Code description
ICD10	W46	Contact with hypodermic needle
ICD10	W46.0	Contact with hypodermic needle
ICD10	W46.1	Contact with contaminated hypodermic needle
ICD10	W46.0XXA	Contact with hypodermic needle, initial encounter
ICD10	W46.0XXD	Contact with hypodermic needle, subsequent encounter
ICD10	W46.0XXS	Contact with hypodermic needle, sequela
ICD10	W46.1XXA	Contact with contaminated hypodermic needle, initial encounter
ICD10	W46.1XXD	Contact with contaminated hypodermic needle, subsequent encounter
ICD10	W46.1XXS	Contact with contaminated hypodermic needle, sequela
ICD10	Z29.9	Unspecified prophylaxis

Table S23: Codes used to identify gonococcal infection.

Code type	Code	Code description	Count[1] (%)
ICD10	A54	Gonococcal infection	12 (0.0)
ICD10	A54.0	Gonococcal infection of lower genitourinary tract without periurethral or accessory gland abscess	1 (0.0)
ICD10	A54.00	Gonococcal infection of lower genitourinary tract	4228 (11.3)
ICD10	A54.01	Gonococcal cystitis and urethritis	3316 (8.9)
ICD10	A54.02	Gonococcal vulvovaginitis	21 (0.1)
ICD10	A54.03	Gonococcal cervicitis	33 (0.1)
ICD10	A54.09	Other gonococcal infection of lower genitourinary tract	544 (1.5)
ICD10	A54.1	Gonococcal infection of lower genitourinary tract with periurethral and accessory gland abscess	158 (0.4)
ICD10	A54.2	Gonococcal pelviperitonitis and other gonococcal genitourinary infection	0 (0.0)
ICD10	A54.21	Gonococcal infection of kidney and ureter	51 (0.1)
ICD10	A54.22	Gonococcal prostatitis	262 (0.7)
ICD10	A54.23	Gonococcal infection of other male genital organs	508 (1.4)
ICD10	A54.24	Gonococcal female pelvic inflammatory disease	13 (0.0)
ICD10	A54.29	Other gonococcal genitourinary infections	243 (0.7)
ICD10	A54.4	Gonococcal infection of musculoskeletal system	0 (0.0)
ICD10	A54.40	Gonococcal infection of musculoskeletal system, unspecified	13 (0.0)
ICD10	A54.41	Gonococcal spondylopathy	10 (0.0)
ICD10	A54.42	Gonococcal arthritis	420 (1.1)
ICD10	A54.43	Gonococcal osteomyelitis	58 (0.2)
ICD10	A54.49	Gonococcal infection of other musculoskeletal tissue	74 (0.2)
ICD10	A54.5	Gonococcal pharyngitis	3771 (10.1)
ICD10	A54.6	Gonococcal infection of anus and rectum	2920 (7.8)
ICD10	A54.8	Other gonococcal infections	0 (0.0)
ICD10	A54.81	Gonococcal meningitis	9 (0.0)
ICD10	A54.82	Gonococcal brain abscess	36 (0.1)
ICD10	A54.83	Gonococcal heart infection	16 (0.0)
ICD10	A54.84	Gonococcal pneumonia	42 (0.1)
ICD10	A54.85	Gonococcal peritonitis	7 (0.0)
ICD10	A54.86	Gonococcal sepsis	120 (0.3)
ICD10	A54.89	Other gonococcal infections	332 (0.9)
ICD10	A54.9	Gonococcal infection	20127 (53.9)
ICD10	O98.211	Gonorrhea complicating pregnancy, first trimester	1 (0.0)
ICD10	O98.212	Gonorrhea complicating pregnancy, second trimester	1 (0.0)
ICD10	O98.213	Gonorrhea complicating pregnancy, third trimester	0 (0.0)
ICD10	O98.219	Gonorrhea complicating pregnancy, unspecified trimester	2 (0.0)
ICD10	O98.22	Gonorrhea complicating childbirth	0 (0.0)
ICD10	O98.23	Gonorrhea complicating the puerperium	24 (0.1)

[1]Counts reflect totals prior to the rollup of codes into distinct episodes; thus, a single infection event may be represented by multiple diagnosis codes.

Table S24: Codes used to identify chlamydia infection.

Code type	Code	Code description	Count[1] (%)
ICD10	A55	Chlamydial lymphogranuloma	495 (1.0)
ICD10	A56.00	Chlamydial infection of lower genitourinary tract	3208 (6.5)
ICD10	A56.01	Chlamydial cystitis and urethritis	6152 (12.4)
ICD10	A56.02	Chlamydial vulvovaginitis	45 (0.1)
ICD10	A56.09	Other chlamydial infection of lower genitourinary tract	698 (1.4)
ICD10	A56.11	Chlamydial female pelvic inflammatory disease	7 (0.0)
ICD10	A56.19	Other chlamydial genitourinary infection	1514 (3.1)
ICD10	A56.2	Chlamydial infection of genitourinary tract, unspecified	2609 (5.3)
ICD10	A56.3	Chlamydial infection of anus and rectum	2334 (4.7)
ICD10	A56.4	Chlamydial infection of pharynx	378 (0.8)
ICD10	A56.8	Sexually transmitted chlamydial infection of other sites	2117 (4.3)
ICD10	A74.8	Other chlamydial diseases	0 (0.0)
ICD10	A74.81	Chlamydial peritonitis	22 (0.0)
ICD10	A74.9	Chlamydial infection, unspecified	30124 (60.6)

[1]Counts reflect totals prior to the rollup of codes into distinct episodes; thus, a single infection event may be represented by multiple diagnosis codes.

Table S25: Codes used to identify syphilis infection.

Code type	Code	Code description	Count[1] (%)
ICD10	A51	Early syphilis	2 (0.0)
ICD10	A51.0	Primary genital syphilis	2953 (3.4)
ICD10	A51.1	Primary anal syphilis	189 (0.2)
ICD10	A51.2	Primary syphilis of other sites	597 (0.7)
ICD10	A51.3	Secondary syphilis of skin and mucous membranes	0 (0.0)
ICD10	A51.31	Condyloma latum	2579 (3.0)
ICD10	A51.32	Syphilitic alopecia	76 (0.1)
ICD10	A51.39	Other secondary syphilis of skin	1639 (1.9)
ICD10	A51.4	Other secondary syphilis	0 (0.0)
ICD10	A51.41	Secondary syphilitic meningitis	48 (0.1)
ICD10	A51.42	Secondary syphilitic female pelvic disease	0 (0.0)
ICD10	A51.43	Secondary syphilitic oculopathy	593 (0.7)
ICD10	A51.44	Secondary syphilitic nephritis	4 (0.0)
ICD10	A51.45	Secondary syphilitic hepatitis	50 (0.1)
ICD10	A51.46	Secondary syphilitic osteopathy	4 (0.0)
ICD10	A51.49	Gonococcal infection of eye, unspecified	3276 (3.8)
ICD10	A51.5	Early syphilis latent	2675 (3.1)
ICD10	A51.9	Early syphilis unspecified	1345 (1.6)
ICD10	A52	Late syphilis	1 (0.0)
ICD10	A52.0	Cardiovascular and cerebrovascular syphilis	0 (0.0)
ICD10	A52.00	Cardiovascular syphilis unspecified	40 (0.1)
ICD10	A52.01	Gonococcal infection of musculoskeletal system, unspecified	51 (0.1)
ICD10	A52.02	Syphilitic aortitis	35 (0.0)
ICD10	A52.03	Syphilitic endocarditis	81 (0.1)
ICD10	A52.04	Syphilitic cerebral arteritis	3 (0.0)
ICD10	A52.05	Other cerebrovascular syphilis	51 (0.1)
ICD10	A52.06	Other syphilitic heart involvement	39 (0.1)
ICD10	A52.09	Other cardiovascular syphilis	4 (0.0)
ICD10	A52.1	Symptomatic neurosyphilis	0 (0.0)
ICD10	A52.10	Symptomatic neurosyphilis, unspecified	284 (0.3)
ICD10	A52.11	Tabes dorsalis	469 (0.6)
ICD10	A52.12	Other cerebrospinal syphilis	45 (0.1)
ICD10	A52.13	Late syphilitic meningitis	141 (0.2)
ICD10	A52.14	Late syphilitic encephalitis	6 (0.0)
ICD10	A52.15	Late syphilitic neuropathy	161 (0.2)
ICD10	A52.17	General paresis	560 (0.7)
ICD10	A52.19	Other symptomatic neurosyphilis	269 (0.3)
ICD10	A52.2	Asymptomatic neurosyphilis	171 (0.2)
ICD10	A52.3	Neurosyphilis, unspecified	4323 (5.0)
ICD10	A52.7	Other symptomatic late syphilis	0 (0.0)
ICD10	A52.71	Late syphilitic oculopathy	734 (0.9)
ICD10	A52.72	Syphilis of lung and bronchus	5 (0.0)
ICD10	A52.73	Symptomatic late syphilis of other respiratory organs	3 (0.0)
ICD10	A52.74	Syphilis of liver and other viscera	44 (0.1)
ICD10	A52.75	Syphilis of kidney and ureter	14 (0.0)
ICD10	A52.76	Other genitourinary symptomatic late syphilis	18 (0.0)
ICD10	A52.77	Syphilis of bone and joint	11 (0.0)
ICD10	A52.78	Syphilis of other musculoskeletal tissue	30 (0.0)
ICD10	A52.79	Other symptomatic late syphilis	140 (0.2)
ICD10	A52.8	Late syphilis latent	3800 (4.4)
ICD10	A52.9	Late syphilis unspecified	1006 (1.2)
ICD10	A53	Other and unspecified syphilis	12 (0.0)
ICD10	A53.0	Latent syphilis, unspecified as early or late	10759 (12.5)
ICD10	A53.9	Syphilis, unspecified	46650 (54.2)
ICD10	O98.1	Syphilis complicating pregnancy, childbirth and the puerperium	0 (0.0)
ICD10	O98.11	Syphilis complicating pregnancy	0 (0.0)
ICD10	O98.111	Syphilis complicating pregnancy, first trimester	0 (0.0)
ICD10	O98.112	Syphilis complicating pregnancy, second trimester	3 (0.0)
ICD10	O98.113	Syphilis complicating pregnancy, third trimester	2 (0.0)
ICD10	O98.119	Syphilis complicating pregnancy, unspecified trimester	12 (0.0)
ICD10	O98.12	Syphilis complicating childbirth	0 (0.0)
ICD10	O98.13	Syphilis complicating the puerperium	0 (0.0)

[1]Counts reflect totals prior to the rollup of codes into distinct episodes; thus, a single infection event may be represented by multiple diagnosis codes.

Table S26: Healthcare Common Procedure Coding System (HCPCS) codes used to identify injectable antibiotics related to sexually transmitted infections.

Code type	Code	Code description	Count[1] (%)
HCPCS	J0456	Injection, azithromycin, 500 mg	99 (0.6)
HCPCS	J0558	Injection, penicillin g benzathine and penicillin g procaine, 100,000 units	230 (1.5)
HCPCS	J0561	Injection, penicillin g benzathine, 100,000 units	6723 (42.8)
HCPCS	J0696	Injection, ceftriaxone sodium, per 250 mg	7841 (49.9)
HCPCS	J0697	Injection, sterile cefuroxime sodium, per 750 mg	2 (0.0)
HCPCS	J0698	Injection, cefotaxime sodium, per gm	2 (0.0)
HCPCS	J0715	Injection, ceftizoxime sodium, per 500 mg	6 (0.0)
HCPCS	J0744	Injection, ciprofloxacin for intravenous infusion, 200 mg	0 (0)
HCPCS	J1580	Injection, garamycin, gentamicin, up to 80 mg	55 (0.4)
HCPCS	J2510	Injection, penicillin g procaine, aqueous, up to 600,000 units	11 (0.1)
HCPCS	J2540	Injection, penicillin g potassium, up to 600,000 units	738 (4.7)

[1]Counts reflect the frequency of each injection within periods where antibiotic treatment was considered to be STI-related, defined as between 3 days before to 3 days after diagnoses of syphilis or gonorrhea, or between 7 days before to 3 days after diagnoses of chlamydia.

Table S27: Oral and injection antibiotics with first- or second-line classification used to treat sexually transmitted infections in the United States 2017 to 2019.

Antibiotic Class	Antibiotic Name	Targeted condition					
		Gonorrhea[1]		*Chlamydia*[2]		*Syphilis*	
		First-line	Second-line	First-line	Second-line	First-line	Second-line
Macrolide	Azithromycin	x	x	x	x		
Macrolide	Azithromycin Dihydrate	x	x	x	x		
Cephalosporin	Cefixime	x	x				
Cephalosporin	Cefotaxime Sodium	x	x				
Quinolone	Ciprofloxacin		x				
Quinolone	Ciprofloxacin Hydrochloride		x				
Quinolone	Ciprofloxacin/Ciprofloxacin Hydrochloride		x				
Quinolone	Levofloxacin		x				
Tetracycline	Doxycycline	x	x	x	x	x	x
Tetracycline	Doxycycline Hyclate	x	x	x	x	x	x
Aminoglycoside	Gentamicin Sulfate		x				
Aminoglycoside	Gentamicin Sulfate/Sodium Chloride		x				
Penicillin	Penicillin G Benzathine					x	x
Penicillin	Penicillin G Benzathine/Penicillin G Procaine					x	x
Penicillin	Penicillin G Potassium					x	x
Penicillin	Penicillin G Potassium/Sodium Chloride					x	x
Penicillin	Penicillin G Procaine					x	x
Penicillin	Penicillin G Sodium					x	x

1; Antibiotics used to treat confirmed gonorrhea infection with and without known co-infection with chlamydia.
2: Antibiotics used to treat chlamydia infection without gonorrhea co-infection.

Table S28: Estimated annual rates of new or casual unprotected anal sex partnerships among MSM according to risk characteristics.

Study for risk parameterization	Stratum	Number of partners, median (interquartile range), by prior-year history of sexually transmitted infections			
		Any history	*≥1 STI diagnosis*	*≥2 STI diagnoses*	*≥3 STI diagnoses*
Jin et al., 2007[8] (primary analysis)					
	All MSM	2 (1-5)	4 (1-15)	12 (3-27)	22 (10-39)
	MSM using PrEP	6 (2-17)	13 (4-29)	22 (10-38)	27 (15-48)
	MSM with HIV	3 (1-8)	6 (2-23)	18 (5-42)	29 (13-59)
Grov et al., 2016[7]					
	All MSM	2 (1-5)	2 (1-6)	2 (1-10)	3 (1-22)
	MSM using PrEP	6 (2-17)	7 (3-22)	11 (3-34)	21 (5-63)
	MSM with HIV	3 (1-8)	3 (1-11)	4 (1-23)	12 (2-75)
Janulis et al., 2023[9]					
	All MSM	2 (1-5)	11 (3-29)	37 (19-65)	62 (38-97)
	MSM using PrEP	6 (2-17)	22 (9-44)	44 (25-70)	64 (41-95)
	MSM with HIV	3 (1-8)	18 (5-46)	52 (28-88)	80 (50-120)

STI: Sexually-transmitted infection (gonorrhea, chlamydia, syphilis).
Citations for included studies (7-9) are listed in the supplemental references.

Supplemental references

1. Mann LM, Le Guillou A, Goodreau SM, et al. Correlations between community-level HIV preexposure prophylaxis coverage and individual-level sexual behaviors among United States MSM. *AIDS* 2022;36(14):2015-2023.

2. Weiss KM, Goodreau SM, Morris M, et al. Egocentric sexual networks of men who have sex with men in the United States: Results from the ARTnet study. *Epidemics* 2020; 30:100386.

3. Zlotorzynska M, Sullivan P, Sanchez T. The annual American Men's Internet Survey of behaviors of men who have sex with men in the United States: 2016 key indicators report. *JMIR Public Health Surveill*. 2019;5(1):e11313. doi:10.2196/11313.

4. Rosenberg ES, Grey JA, Sanchez TH, Sullivan PS. Rates of prevalent HIV infection, prevalent diagnoses, and new diagnoses among men who have sex with men in US States, Metropolitan Statistical Areas, and Counties, 2012-2013. *JMIR Public Health Surveill*. 2016;2(1):e5684.

5. US Centers for Disease Control & Prevention. Sexually Transmitted Infections Surveillance, 2023. Available from: https://www.cdc.gov/sti-statistics/annual/index.html.

6. Raymond HF, Rebchook G, Curotto A, et al. Comparing internet-based and venue-based methods to sample MSM in the San Francisco Bay area. *AIDS Behav* 2010;14(1):218-224. doi:10.1007/s10461-009-9521-6.

7. Grov C, Cain D, Rendina HJ, Ventuneac A, Parsons JT. Characteristics associated with urethral and rectal gonorrhea and chlamydia diagnoses in a US national sample of gay and bisexual men: results From the One Thousand Strong panel. *Sex Transm Dis* 2016; 43(3):165.

8. Jin F, Prestage GP, Mao L, et al. Incidence and risk factors for urethral and anal gonorrhoea and chlamydia in a cohort of HIV-negative homosexual men: the Health in Men Study. *Sex Transm Infect* 2007; 83(2):113-119.

9. Janulis P, Goodreau SM, Morris M, et al. Partnership types and coital frequency as predictors of gonorrhea and chlamydia among young MSM and young transgender women. *Int J STD AIDS* 2023; 34(10):694-701.

10. Jasuja GK, de Groot A, Quinn EK, et al. Beyond gender identity disorder diagnosis codes: an examination of additional methods to identify transgender individuals in administrative databases. *Med Care* 2020;58(10):903-911.

11. Garner W, Wilson BM, Beste L, Maier M, Ohl ME, Van Epps P. Gaps in preexposure prophylaxis uptake for HIV prevention in the Veterans Health Administration. *Am J Public Health* 2018;108(S4):S305-S310.

12. Furukawa NW, Smith DK, Gonzalez CJ, et al. Evaluation of algorithms used for PrEP surveillance using a reference population from New York City, July 2016–June 2018. *Public Health Rep* 2020; 135(2):202-210.

13. Lillis R, Kuritzky L, Huynh Z, et al. Outpatient sexually transmitted infection testing and treatment patterns in the United States: a real-world database study. *BMC Infect Dis*. 2023;23(1):1-10. doi:10.1186/s12879-023-08434-2.
