# Supplementary material for: Bacterial sexually transmitted infections and related antibiotic use among individuals eligible for doxycycline post-exposure prophylaxis in the United States
Source: Nat Commun. 2025 Oct 16;16:9206. doi: 10.1038/s41467-025-64261-w (PMC12532492; doi:10.1038/s41467-025-64261-w)
Supplement: Supplementary file 2 — Reporting Summary [file 41467_2025_64261_MOESM2_ESM.pdf]

nature portfolio

Corresponding author(s): Lewnard

Last updated by author(s): 9/2/2025

# Reporting Summary

Nature Portfolio wishes to improve the reproducibility of the work that we publish. This form provides structure for consistency and transparency in reporting. For further information on Nature Portfolio policies, see our Editorial Policies and the Editorial Policy Checklist.

## Statistics

For all statistical analyses, confirm that the following items are present in the figure legend, table legend, main text, or Methods section.

| n/a | Confirmed | |
|---|---|---|
| ☐ | ☒ | The exact sample size (*n*) for each experimental group/condition, given as a discrete number and unit of measurement |
| ☒ | ☐ | A statement on whether measurements were taken from distinct samples or whether the same sample was measured repeatedly |
| ☐ | ☒ | The statistical test(s) used AND whether they are one- or two-sided<br>*Only common tests should be described solely by name; describe more complex techniques in the Methods section.* |
| ☐ | ☒ | A description of all covariates tested |
| ☒ | ☐ | A description of any assumptions or corrections, such as tests of normality and adjustment for multiple comparisons |
| ☐ | ☒ | A full description of the statistical parameters including central tendency (e.g. means) or other basic estimates (e.g. regression coefficient) AND variation (e.g. standard deviation) or associated estimates of uncertainty (e.g. confidence intervals) |
| ☒ | ☐ | For null hypothesis testing, the test statistic (e.g. *F*, *t*, *r*) with confidence intervals, effect sizes, degrees of freedom and *P* value noted<br>*Give P values as exact values whenever suitable.* |
| ☒ | ☐ | For Bayesian analysis, information on the choice of priors and Markov chain Monte Carlo settings |
| ☒ | ☐ | For hierarchical and complex designs, identification of the appropriate level for tests and full reporting of outcomes |
| ☐ | ☒ | Estimates of effect sizes (e.g. Cohen's *d*, Pearson's *r*), indicating how they were calculated |

*Our web collection on statistics for biologists contains articles on many of the points above.*

## Software and code

Policy information about availability of computer code

| | |
|---|---|
| Data collection | This study involved secondary use of pre-existing data. No custom software/tools/algorithms/packages were used in data collection. |
| Data analysis | Analyses used R (version 4.5.0; R Foundation for Statistical Computing, Vienna, Austria). Analysis code is available from: https://github.com/joelewnard/doxyPEP. |

For manuscripts utilizing custom algorithms or software that are central to the research but not yet described in published literature, software must be made available to editors and reviewers. We strongly encourage code deposition in a community repository (e.g. GitHub). See the Nature Portfolio guidelines for submitting code & software for further information.

## Data

Policy information about availability of data

All manuscripts must include a data availability statement. This statement should provide the following information, where applicable:

- Accession codes, unique identifiers, or web links for publicly available datasets
- A description of any restrictions on data availability
- For clinical datasets or third party data, please ensure that the statement adheres to our policy

The study population for this retrospective cohort study using the MerativeTM MarketScan® Research Databases consisted of commercially-insured males and transgender individuals aged 18-64 years at any time between 1 January, 2017 and 31 December, 2019.  Raw data from the MarketScan insurance claims databases are available for licensed users. A user license could be obtained by following the instructions at https://marketscan.truvenhealth.com/marketscanportal/.

## Research involving human participants, their data, or biological material

Policy information about studies with human participants or human data. See also policy information about sex, gender (identity/presentation), and sexual orientation and race, ethnicity and racism.

| | |
|---|---|
| Reporting on sex and gender | The study population for this retrospective cohort study using the MerativeTM MarketScan® Research Databases consisted of commercially-insured males and transgender individuals, defined on the basis of diagnosis or procedure codes indicating receipt of gender-affirming care (Table S17). Our Discussion section acknowledges the limitation that claims data offer limited sensitivity in representing sexual and gender-minority populations. |
| Reporting on race, ethnicity, or other socially relevant groupings | Our analysis does not address race/ethnicity, which are not uniformly measured in claims data. While not restricted to men who have sex with men (MSM), the study population is likely comprised largely of this group. Although MSM and non-MSM cannot be readily distinguished from administrative claims data, MSM comprise the vast majority of US males using HIV pre-exposure prophylaxis and account for 87% of US males living with HIV. These were the two primary enrollment criteria for analyses. We also defined a subgroup of individuals with history of bacterial sexually transmitted infection diagnoses; we likewise expect MSM to be over-represented within this stratum. |
| Population characteristics | We present descriptive characteristics of the study population as ranges of rates or proportions across cohorts eligible for analysis in 2016, 2017, and 2018 in Table S1. From 2017-2019, eligible cohorts of PrEP recipients and PLWH increased in size from 10,679-15,384 individuals and from 8,095-11,011 individuals, respectively. Within each year, >99% of individuals eligible for analyses were male, and >90% had no history of bacterial sexually transmitted infections in the previous year. Median ages were between 35-44 years for individuals receiving HIV pre-exposure prophylaxis and >45 years for those living with HIV. |
| Recruitment | This analysis comprised members of commercial health insurance plans represented in the MerativeTM MarketScan® Research Databases; no active recruitment for the study was undertaken. |
| Ethics oversight | These analyses of deidentified insurance claims data were considered exempt from review by the University of Alabama, Birmingham Institutional Review Board and the Committee for the Protection of Human Subjects at the University of California, Berkeley. |

Note that full information on the approval of the study protocol must also be provided in the manuscript.

# Field-specific reporting

Please select the one below that is the best fit for your research. If you are not sure, read the appropriate sections before making your selection.

☐ Life sciences ☒ Behavioural & social sciences ☐ Ecological, evolutionary & environmental sciences

For a reference copy of the document with all sections, see nature.com/documents/nr-reporting-summary-flat.pdf

# Behavioural & social sciences study design

All studies must disclose on these points even when the disclosure is negative.

| | |
|---|---|
| Study description | We undertook a retrospective observational cohort study using quantitative data. The study cohort comprised individuals covered by health plans represented in the MerativeTM MarketScan® Research Databases, a US-wide collection of administrative and commercial healthcare data from adjudicated claims across multiple commercial insurance plans. Outcomes were measured via diagnoses, prescriptions, and procedures recorded via insurance claim reimbursements. |
| Research sample | The population consisted of commercially-insured males and transgender individuals aged 18-64 years at any time between 1 January, 2017 and 31 December, 2019 whose health plans were represented in the MerativeTM MarketScan® Research Databases, a US-wide collection of administrative and commercial healthcare data from adjudicated claims across multiple commercial insurance plans collectively representing over 50 million enrollees.<br><br>We included time-at-risk for each individual following any 12-month period (extending as early as 1 January, 2016) during which they were continuously enrolled in both medical and prescription coverage (no lapse >60 days) and filled ≥1 prescription. The population eligible for the study included individuals likely be prioritized for doxyPEP implementation, including people living with HIV (PLWH), recipients of HIV pre-exposure prophylaxis (PrEP), and those with prior STI diagnoses. We used prior-year healthcare utilization (measured from 2016-2018) to group individuals into these categories during each calendar year. We required ≥1 prescription fill to ensure the data encompassed individuals who actively used both medical and prescription insurance plans. In total, follow-up for the estimates plotted encompasses 38,543 person-years at risk among PrEP recipients, 29,228 person-years at risk among PLWH, and 19,918 person-years at risk among people with prior STI diagnosis history, summed across all years of follow-up (2017-2019). The number of unique individuals included each year, in each eligibility stratum, is as follows:<br>PLWH: 8,095 (2017), 10,122 (2018), 11,011 (2019)<br>PrEP recipients: 10,679 (2017), 12,480 (2018), 15,384 (2019)<br>STI history: 5,621 (2017), 6,688 (2018), 7,609 (2019)<br><br>Characteristics of the full population of individuals eligible to receive doxyPEP in the US are not known, largely because these populations represent sexual or gender minority groups and comprise "hidden" populations. While the MarketScan commercial claims cohort broadly represents a convenience sample of individuals with commercial insurance plans, the specific eligibility criteria |

| | |
|---|---|
| | for this study aligned the population included in analyses with those eligible to receive doxyPEP. Although persons already receiving PrEP and PLWH may not encompass all individuals who could benefit from doxyPEP, we considered these populations to represent those most likely to adopt doxyPEP given their existing linkage to preventive sexual health services.<br><br>While most (>60%) PLWH were aged ≥45 years, PrEP recipients represented a younger sample (46-54% aged 25-44 years), and most individuals with history of STI diagnoses were aged <35 years. Below 1% of individuals were identified as transgender, although this status may be under-counted in claims data due to the reliance on diagnosis codes and procedures to identify transgender individuals. |
| Sampling strategy | All individuals meeting eligibility criteria were included in analyses; there was no pre-specified sample size. The MarketScan commercial claims cohort broadly represents a convenience sample of individuals with commercial insurance plans. As our analyses did not include inferential comparisons across groups, a minimum a prior sample size determination was not required to assess statistical power for primary or secondary hypotheses. With >10,000 person-years at risk represented in each of the primary risk strata analyzed, the available sample size was expected to be sufficient to characterize incidence of even rare events within acceptable degrees of precision. |
| Data collection | We made secondary use of existing data from the MerativeTM MarketScan Research Databases. Briefly, data encompass all diagnoses, prescriptions, and procedures submitted for insurance reimbursement. Diagnoses are coded using the International Classification of Diseases, Tenth Revision (ICD-10) codes; prescriptions are recorded using National Drug Codes (NDCs); and procedures are coded via Healthcare Common Procedure Coding System (HCPCS) codes. We enumerate codes used for defining study outcomes and exposures/covariates in Tables S18-S22 and Tables S26-S27. Researchers were not blind to study participants' exposure; this study did not involve hypothesis testing or other inferential comparisons of outcomes between distinct exposure groups. |
| Timing | Our analysis period encompassed 1 January, 2017 and 31 December, 2019 to mitigate impacts of transient behavioral changes associated with emergency phases of the COVID-19 pandemic and subsequent clade II mpox epidemic. |
| Data exclusions | No data were excluded from analyses. Individual eligibility criteria for analyses are described above ("Research sample"). |
| Non-participation | N/A; the study did not involve primary recruitment. |
| Randomization | No randomization was performed. This study did not involve allocation into, or comparison across, distinct exposure groups; rather, we present descriptive characteristics (incidence rates) of the study outcome in several groups meeting existing or potential eligibility criteria for doxyPEP receipt. We defined exposure groups based on individuals' medical history, including criteria used to define eligibility for doxyPEP in existing clinical guidelines (HIV infection status, receipt of HIV pre-exposure prophylaxis, and STI diagnosis history). As all analyses were descriptive, no analyses included covariates for adjustment. |

# Reporting for specific materials, systems and methods

We require information from authors about some types of materials, experimental systems and methods used in many studies. Here, indicate whether each material, system or method listed is relevant to your study. If you are not sure if a list item applies to your research, read the appropriate section before selecting a response.

## Materials & experimental systems

| n/a | Involved in the study |
|---|---|
| ☒ | ☐ Antibodies |
| ☒ | ☐ Eukaryotic cell lines |
| ☒ | ☐ Palaeontology and archaeology |
| ☒ | ☐ Animals and other organisms |
| ☐ | ☒ Clinical data |
| ☒ | ☐ Dual use research of concern |
| ☒ | ☐ Plants |

## Methods

| n/a | Involved in the study |
|---|---|
| ☒ | ☐ ChIP-seq |
| ☒ | ☐ Flow cytometry |
| ☒ | ☐ MRI-based neuroimaging |

## Clinical data

Policy information about clinical studies
All manuscripts should comply with the ICMJE guidelines for publication of clinical research and a completed CONSORT checklist must be included with all submissions.

| | |
|---|---|
| Clinical trial registration | N/A (not a clinical trial) |
| Study protocol | No pre-specified protocol was written for this observational study. |
| Data collection | Diagnoses, prescriptions, and procedures were abstracted from insurance claims, as represented in the MerativeTM MarketScan® Research Databases. |
| Outcomes | Study outcomes were diagnoses and class-specific antibiotic fill-days for bacterial STIs (chlamydia, gonorrhea, and syphilis infections). We identified oral antibiotic prescriptions using NDCs with therapeutic classifications of 4 or 6-20, and injectable antibiotics via |

Healthcare Common Procedure Coding System (HCPCS) codes (Table S26). An antibiotic fill-day was defined as a day with ≥1 oral antibiotic fill or antibiotic injection. As syphilis treatment strategies frequently involve injections over multiple days, multiple fill-days could result from a single syphilis episode. We defined STI-associated fills with first- or second-line antibiotics (Table S27) as those occurring within 3 days before or after syphilis and gonorrhea diagnoses, and within 7 days before to 3 days after a chlamydia diagnosis (allowing a long period to capture antibiotics received prior to chlamydia diagnoses to account for presumptive treatment during gonorrhea co-infection). Chlamydia mono-infections were those occurring without accompanying gonorrhea infections within periods 3 days before to 7 days diagnosis.

## Plants

| | |
|---|---|
| Seed stocks | N/A |
| Novel plant genotypes | N/A |
| Authentication | N/A |
